# Ophiostomatoid fungi associated with mites phoretic on bark beetles in Qinghai, China

**DOI:** 10.1186/s43008-020-00037-9

**Published:** 2020-07-30

**Authors:** Runlei Chang, Tuan A. Duong, Stephen J. Taerum, Michael J. Wingfield, XuDong Zhou, Z. Wilhelm de Beer

**Affiliations:** 1 Department of Biochemistry, Genetics & Microbiology, Forestry and Agricultural Biotechnology Institute (FABI), University of Pretoria, Pretoria, 0002 South Africa; 2 College of Life Sciences, Shandong Normal University, Jinan, 250014 China

**Keywords:** Spruce, Symbiosis, Ascomycetes, *Scolytinae*, Three new taxa

## Abstract

Bark beetle galleries are complex ecosystems where many microbes and other arthropods co-exist with the beetles. Fungi isolated from these galleries are often referred to as ‘beetle associates’, but the nature of these associations are poorly understood. The possibility that many of these fungi might in fact be mite associates is often overlooked. Several recent studies explored the diversity of fungi from conifer-infesting bark beetles and their galleries in China, but only one study considered phoretic mites and their fungi from conifer-infesting bark beetles in Yunnan, southwestern China. We studied the mites and fungi from galleries of four spruce-infesting bark beetle species in the high altitude forests of Qinghai province, western China. Mites were identified based on morphological characteristics, and fungi based on DNA sequences of four gene regions. In total, 173 mite individuals were collected belonging to 18 species in 11 genera. A total of 135 fungal isolates were obtained from the mites, representing 14 taxa from the *Ophiostomatales.* The most frequently isolated fungus was *Ophiostoma nitidum*, which represented 23.5% of the total isolates. More fungal species were found from fewer mites and bark beetle species than from the study in Yunnan. Although we could not elucidate the exact nature of interactions between mites and their fungi, our results re-enforce that these organisms should not be ignored in pest risk assessments of bark beetles, that often focus only on the beetles and their fungi. Three new species are described: *Grosmannia zekuensis*, *O. manchongi,* and *O. kunlunense* spp. nov., and our data revealed that *O. typographi*, recently described from China, is a synonym of *O. ainoae*.

## INTRODUCTION

Mites (*Arachnida*, *Acari*) are commonly associated with bark beetles (*Insecta*, *Coleoptera*, *Scolytinae*) and their galleries (Hofstetter et al. 2015). Because of their small size, these mite species rely on the bark beetles for dispersal between plant hosts (Hofstetter et al. 2013). To date, about 270 mite species have been identified as associates of a limited number of bark beetle species, and many more are likely to be discovered (Hofstetter et al. 2013, 2015).

The ecological roles of very few bark beetle-associated mites are well understood, but the different classes of feeding behavior (Hofstetter et al. 2013) suggests that their impacts in gallery ecosystems could be broad. Some of these mites, such as *Pyemotes dryas*, predate upon or parasitize the eggs and early larval instars of bark beetles (Wegensteiner et al. 2015) and thus have a direct effect on population dynamics of the beetles. However, the impacts of many mites on bark beetle behavior and population growth are indirect. A few species are known to be predators of nematodes, while many of the mite species are omnivores and feed on fungi, nematodes and dead arthropods in the galleries. Other species are exclusively mycetophagous, utilizing fungi as their only source of nutrition (Hofstetter et al. 2013).

It appears that some mycetophagous mites have preference for one or a few specific species of fungi. One such an example is *Tarsonemus krantzi,* associated with the southern pine beetle, *Dendroctonus frontalis*, that vector and feeds on *Ophiostoma minus* (Lombardero et al. 2000). Other mites such as *Histiogaster* spp. are generalists that can feed on several different fungal species (Hofstetter and Moser 2014). The most common fungi associated with bark beetles and their mites are the ophiostomatoid fungi (Hofstetter and Moser 2014). This is a polyphyletic group of fungi that includes several genera in *Microascales* and *Ophiostomatales*, that are characterized by spores produced in sticky droplets to facilitate dispersal by bark beetles and mites (De Beer et al. 2013). Many bark beetles have specialized structures known as mycangia in which to carry the spores of these fungi (Six 2012). In the case of mites, most fungi appear to be vectored on their exterior surfaces (Moser et al. 1989b), but some mite species have evolved specialized structures known as, sporothecae, to ensure the successful transmission of specific fungal symbionts between hosts (Moser 1985).

Although the impact of most fungi on the fitness of the mites or their beetle vectors remains largely unknown, the three-way interaction between beetles, mites and fungi, has been elucidated in the case of the southern pine beetle, *Dendroctonus frontalis*, and its associated *Tarsonemus* mites. The larvae of *D. frontalis* are obligately dependent on two fungal species, *Ceratocystiopsis ranaculosus* and *Entomocorticium* sp. A, for nutrition (Ayres et al. 2000). These fungi are outcompeted by *Ophiostoma minus* (Barras 1970; Bridges 1983; Goldhammer et al. 1990), the nutritional fungal mutualist of *Tarsonemus* mites, when the mites are present in the galleries of *D. frontalis* (Hofstetter et al. 2013). As a result of these interactions, large populations of *Tarsonemus* mites can suppress the reproduction of *D. frontalis* (Hofstetter et al. 2006).

The relationships between mites and their beetle vectors are generally unspecific, and a single species of mite can use many different beetle species as vector in order to reach its tree hosts (Hofstetter et al. 2013). Similarly, many ophiostomatoid fungi are promiscuous in their association with beetle species, and a single beetle species can vector a number of different fungal species (Kirisits 2004; Linnakoski et al. 2012; Taerum et al. 2013). In addition, one mite species can carry any of a number of ophiostomatoid fungi (Chang et al. 2017). It thus becomes extremely difficult to resolve specificity between any of these organisms. However, where galleries of different bark beetle species might be restricted to different parts of a specific tree and thus not overlap, it has been suggested that mites might facilitate the movement of fungal species between galleries of different beetle species (Chang et al. 2017).

There are several serious tree pathogens amongst the ophiostomatoid fungi. These include the well-known Dutch Elm Disease fungi, *Ophiostoma ulmi* and two varieties of *O. novo-ulmi* (Brasier 1990, 1991). These pathogens are vectored by *Scolytus* bark beetles (Webber 2004), but it has been shown that phoretic mites also vector the pathogens and might contribute to the spore load and the high efficiency of *S. scolytus* in spreading Dutch elm disease (Moser et al. 2010). *Leptographium wingfieldii* is a mildly pathogenic ophiostomatoid fungus vectored by the pine shoot beetle, *Tomicus piniperda*, in its native range in Europe (Solheim 1991). The beetle was first detected in the USA in the early 1990’s, and it was later shown that *L. wingfieldii* was introduced with the beetle, and that the fungus has subsequently became an associate of two native north American beetles, *Dendroctonus valens* and *Ips pini* (Jacobs et al. 2004). Although the mechanism by which *L. wingfieldii* was transferred between the beetle species was not considered (Jacobs et al. 2004), it is likely that mites facilitated the transfer. Such novel associations between ophiostomatoid fungi, bark- or ambrosia beetles and trees, can pose serious threats to both natural and commercial forests, as well as tree crops. The role of mites in the establishment of these associations are poorly studied, most probably underestimated, and can only be understood if their associations with fungi are explored in natural ecosystems.

The taxonomy of *Ophiostomatales* was revised by De Beer & Wingfield (2013) who considered all published ribosomal large subunit (LSU) and internal transcribed spacer (ITS) sequences. They recognized six genera and 18 species complexes in the order, including *Ophiostoma*, *Raffaelea*, *Ceratocystiopsis*, *Fragosphaeria*, *Graphilbum*, and *Leptographium sensu lato*. In a subsequent paper De Beer et al. (2016a) elevated the *S. schenckii* – *O. stenoceras* complex to genus level, and re-instated the name *Sporothrix* for this group. Three smaller, novel genera had also been described recently in the order, *Hawksworthiomyces* (De Beer et al. 2016b), *Aureovirgo* (Van Der Linde et al. 2016) and *Afroraffaelea* (Bateman et al. 2017). For the purpose of the present study, we define *Ophiostoma sensu stricto* in agreement with De Beer et al. (2016a), that then includes the *O. ulmi–, O. piceae–, O. ips–,* and *O. clavatum* species complexes. A number of *Ophiostoma* species group outside *Ophiostoma s. str.*, and their position remain unresolved. For the present they are included next to *Ophiostoma s. str.* in a more loosely defined *Ophiostoma sensu lato. Leptographium s. lat.* incorporates *Leptographium s. str.* and the *Grosmannia penicillata* complex as defined by De Beer and Wingfield (2013) and Yin et al. (2020).

To date, the majority of studies on interactions between fungi, bark beetles and mites have been conducted on *Dendroctonus*, *Ips* and *Dryocoetes* spp. in North America (Klepzig and Hofstetter 2011; Hofstetter et al. 2013, 2015; Hofstetter and Moser 2014), and *Scolytus, Ips* and *Pityokteines* spp. in Europe (Levieux et al. 1989; Moser et al. 1989a, 2005, 2010; Linnakoski et al. 2016b). In southern Africa, ongoing studies focus on the interactions between mites and ophiostomatoid fungi in infructescences of *Protea* spp. (Roets et al. 2007, 2009, 2011). There have been only two studies reporting on the fungal associates of mites form east Asia. In these cases, Moser et al. (1997) reported on the fungal associates of mites on *Ips typographus* in Japan, and Chang et al. (2017) described fungi from mites associated with various conifer-infesting bark beetles in Yunnan, China.

In recent years, 98 ophiostomatoid species, including 49 new species, have been reported in association with bark beetles from China (Table S1) (Lu et al. 2009a, 2009b; Paciura et al. 2010a, 2010b; Zhou et al. 2011, 2013; Taerum et al. 2013; Yin et al. 2015, 2016, 2019, 2020; Wang et al. 2016, 2018, 2019, 2020; Chang et al. 2017, 2019; Liu et al. 2017). The study by Chang et al. (2017) was the first to report ophiostomatoid fungi associated with phoretic mites. They reported 11 species from mites associated with bark beetles infesting *Pinus kesiya*, of which four were described as new species. Three of the studies from China, included isolates from Qinghai province (Yin et al. 2016, 2019, 2020). This province, which is located on the Qinghai–Tibetan Plateau, is one of the world’s biodiversity hotspots because of its diverse landscapes, and complex geological and climatic history (Li et al. 2012). Yin et al. (2016) decribed five new *Ophiostoma* spp. from four spruce-infesting bark beetles, two of which are *Ips* spp. that cause severe damage to spruce trees are in this area (Liu et al. 2008). In addition, two *Leptographium* (Yin et al. 2019) and four *Grosmannia* spp. (Yin et al. 2020) were described from *Polygraphus poligraphus* and *Ips shangrila*, also attacking spruce. Apart from these 11 species, no other ophiostomatoid fungi have been reported from Qinghai. Furthermore, nothing is known regarding the fungal associates of phoretic mites on spruce-infesting beetles in China. In this study, we addressed the following questions: 1) which mite species are associated with spruce-infesting bark beetles in Qinghai, and 2) which species of ophiostomatoid fungi are associated with these mites?

## MATERIAL AND METHODS

### Collection of mites and fungi

A survey was conducted on *Picea crassifolia* and *Picea purpurea* in July 2010, during the flight period of bark beetles, in the Maixiu and Xianmin forest farms in Qinghai province, China. Bark beetle galleries were collected and stored in re-sealable plastic bags at 4 °C until isolations could be made. Living mites were collected from the galleries under a dissecting microscope. Each individual mite was placed on a separate Petri dish containing malt extract agar (MEA, 20 g Difico agar, 20 g Difico BactoTM malt extract [Becton, Dickinson & Company], 1 L deionized water) medium. After the plates were sealed, the mites were allowed to crawl over the plates for 24 h. The mites were then removed and stored in 1.5 mL Eppendorf tubes containing 75% Ethanol for later identification by Dr. E.A. Ueckermann (Plant Protection Research Institute, Agricultural Research Council, South Africa).

The MEA plates were incubated at 20 °C until fungal growth was evident. The hyphal tips of colonies were transferred to fresh MEA plates to obtain pure cultures. All isolates used in this study were deposited into the Culture Collection (CMW) of the Forestry and Agricultural Biotechnology Institute (FABI), University of Pretoria, Pretoria, Republic of South Africa. Isolates representing types of new species were deposited in the culture collection (CBS) of the Westerdijk Fungal Biodiversity Institute, Utrecht, the Netherlands.

### DNA sequencing and phylogenetic analyses

Isolates were grown on 2% MEA medium. DNA was extracted using PrepMan ultra sample preparation reagent (Applied Biosystems, Foster City, CA) following the manufacturer’s recommendations. The internal transcribed spacer regions 1 and 2 (ITS), including the 5.8S region, were amplified using the primers ITS1F and ITS4 (White et al. 1990; Gardes and Bruns 1993), the β-tubulin (*BT*) gene was amplified using the primer pair of Bt2a and Bt2b (Glass and Donaldson 1995), and the elongation factor 1-α (*EF*) gene was amplified using the primer pair of EF2F (Marincowitz et al. 2015) and EF2R (Jacobs et al. 2004). In addition, the nuclear large subunit (LSU) was amplified with the primer pair LR0R and LR5 (Vilgalys and Hester 1990) for fungi that reside in *Leptographium sensu lato*. PCR and sequencing were conducted following the protocols described by Duong et al. (2012).

The sequences obtained with the forward and reverse primers were aligned and contigs constructed using the program Geneious pro v. 7.1.4 (Biomatters, Auckland, New Zealand). All sequences obtained in this study were deposited in GenBank. BLAST searches of the ITS sequences were conducted in NCBI GenBank for preliminary identifications. Based on the BLAST results, sequence data for other markers were separated according to the relevant species complexes. For taxa residing in *Leptographium s. lat.,* the ITS2-LSU regions were used to determine generic placement, unlike the case for *Ophiostoma* spp. where the ITS1-ITS2 regions were used for this purpose. The *BT* and *EF* data sets were analyzed separately for each species complex. Alignments were made using an online version of MAFFT v. 7 with default settings (Katoh and Standley 2013). All aligned sequence datasets were submitted to TreeBase (No. 24829).

Phylogenetic analyses including maximum likelihood (ML), maximum parsimony (MP) and Bayesian inference (BI) were conducted for all datasets. The best substitution models for each data set were determined using jModelTest v. 2.1.6 (Darriba et al. 2012) on the CIPRES Science Gateway v. 3.3 (Miller et al. 2010). ML analyses were conducted using RaxML v. 8.2.4 on the CIPRES Science Gateway v. 3.3 (Stamatakis 2014) with default GTR substitution matrix and 1000 rapid bootstraps. MP analyses were performed using PAUP v. 4.0b10 (Swofford 2002), gaps were treated as a fifth character. BI analyses were conducted using MrBayes v. 3.2.6 (Ronquist et al. 2012) on the CIPRES Science Gateway v. 3.3. Four MCMC chains were run from a random starting tree for 5 million generations and trees were sampled every 100th generation. 25% of trees sampled were discarded as burn-in and the remaining trees were used to construct majority rule consensus trees.

### Growth studies

Mycelium-covered agar plugs were transferred from the actively growing margins of one-week-old cultures and placed at the centers of 90 mm Petri dishes containing 2% MEA. The optimal temperatures for growth were determined at temperatures ranging from 5 to 35 °C at 5 °C intervals and there were three replicate plates for each temperature. Cultures were incubated in the dark. Colony diameters were measured every 2 days until hyphae reached the edges of the Petri dishes, at which point the temperatures for optimum growth were noted.

### Morphological studies

Asexual and/or sexual structures were mounted in lactophenol on glass slides, covered with coverslips and examined with a Zeiss Axioskop 2 Plus compound microscope or a Zeiss Discovery V12 dissection microscope with an Axiocam digital camera (Axiovision 3.1) (München-Hallbergmoos, Germany). Fifty measurements were made for each taxonomically informative structure. The measurements were given in the format (minimum-) mean minus standard deviation-mean plus standard deviation (−maximum).

### Frequency of isolation

The following formula was used to calculate the frequencies of isolation of the ophiostomatoid species: F = (NF/NT) × 100, where F represented the frequency of isolation (%), NT represented the total number of isolates obtained, and NF represented the number of isolates for each particular taxon.

## RESULTS

### Collection of mites and fungi

In total, 173 mite individuals representing 18 mite species residing in 11 genera were collected from galleries of four bark beetle species on two host tree species (Table 1). The bark beetles were *Dendroctonus micans*, *Ips shangrila*, *Ips nitidus* and *Polygraphus poligraphus*. All *D. micans*, *I. nitidus* and *P. poligraphus* were collected from *Pi. crassifolia*, and *I. shangrila* was collected from *Pi. purpurea*. Three mite species were associated with *D. micans*, 13 mite species with *I. nitidus*, five mite species with *I. shangrila*, and three mite species with *P. poligraphus*. Most mite species were found only in the galleries of a single bark beetle species. Exceptions were for *Insectolaelaps* sp. 1, *Uropodoidea* sp. 4, *Uropodoidea* sp. 6, and *Winterschmidtiidae* sp., which were found in the galleries of more than one beetle species (Table S2).

**Table 1:** Mites (*Acari*) collected from bark beetle galleries on spruce in Qinghai in this study

Mite	Family Name	Species Name	NI^**a**^	NI/TN^**b**^	NMCF^**c**^	NMCF/NI^**d**^
M 1	*Acaridae*	*Horstia* sp.	1	0.0061	0	0.000
M 2	*Acaridae*	*Schwiebea wainsteini*	2	0.0121	2	1.000
M 3	*Ascidae*	*Diseius* cf. *ulmi*	3	0.0182	1	0.333
M 4	*Digamasellidae*	*Dendrolaelaps* sp.	1	0.0061	0	0.000
M 5	*Digamasellidae*	*Insectolaelaps* sp. 2	23	0.1394	7	0.304
M 6	*Digamasellidae*	*Insectolaelaps* sp. 1	15	0.0909	7	0.467
M 7	*Ereynetidae*	*Ereynetes* sp.	3	0.0182	2	0.667
M 8	*Erynetidae*	sp.	1	0.0061	0	0.000
M 9	Unknown	sp. 1	6	0.0364	1	0.167
M 10	*Melicharidae*	*Proctolaelaps* nr. *hystrix*	10	0.0606	5	0.500
M 11	*Mesostigmata*	sp. 1	1	0.0061	1	1.000
M 12	*Mesostigmata*	sp. 2	1	0.0061	0	0.000
M 13	*Mesostigmata*	sp. 3	1	0.0061	1	1.000
M 14	*Mesostigmata*	sp. 4	2	0.0121	1	0.500
M 15	Unknown	sp. 2	6	0.0364	1	0.167
M 16	*Pygmephoridae*	*Bakerdania* sp.	5	0.0303	1	0.200
M 17	*Tarsonemidae*	*Tarsonemus* sp.	7	0.0424	1	0.143
M 18	*Uropodoidea*	sp. 4	35	0.2121	13	0.371
M 19	*Uropodoidea*	sp. 5	1	0.0061	0	0.000
M 20	*Uropodoidea*	sp. 6	35	0.2121	9	0.257
M 21	*Uropodoidea*	sp. 7	1	0.0061	1	1.000
M 22	*Uropodoidea*	sp. 8	1	0.0061	0	0.000
M 23	*Winterschmidtiidae*	sp.	11	0.0667	10	0.909
M 24	*Zerconidae*	*Zercon* sp.	1	0.0061	1	1.000
Total			173		65	0.376

^a^*NI* Number of mite individuals

^b^*NI/TN* Number of mite individuals/Total number of mite individuals

^c^*NMCF* Number of mites carrying fungi

^d^*NMCF/NI* Number of mites carrying fungi/ Number of mite individuals

In total, 135 ophiostomatoid fungal isolates were obtained from 65 mite individuals (Table S3). Seventy isolates were collected from 13 mite species in galleries of *I. nitidus*, 33 isolates were collected from three mite species in galleries of *D. micans*, 19 isolates were collected from five mite species in galleries of *I. shangrila,* and 13 isolates were collected from three mite species in *P. poligraphus*. Twenty-eight isolates were collected from one mite species in the family *Winterschmidtiidae* and 23 isolates collected from *Uropodoidea* sp. 4. More than 10 isolates were collected from each of *Insectolaelaps* sp. 2, *Insectolaelaps* sp. 1 and *Uropodoidea* sp. 6.

### DNA sequencing and phylogenetic analysis

**Fig. 1:**
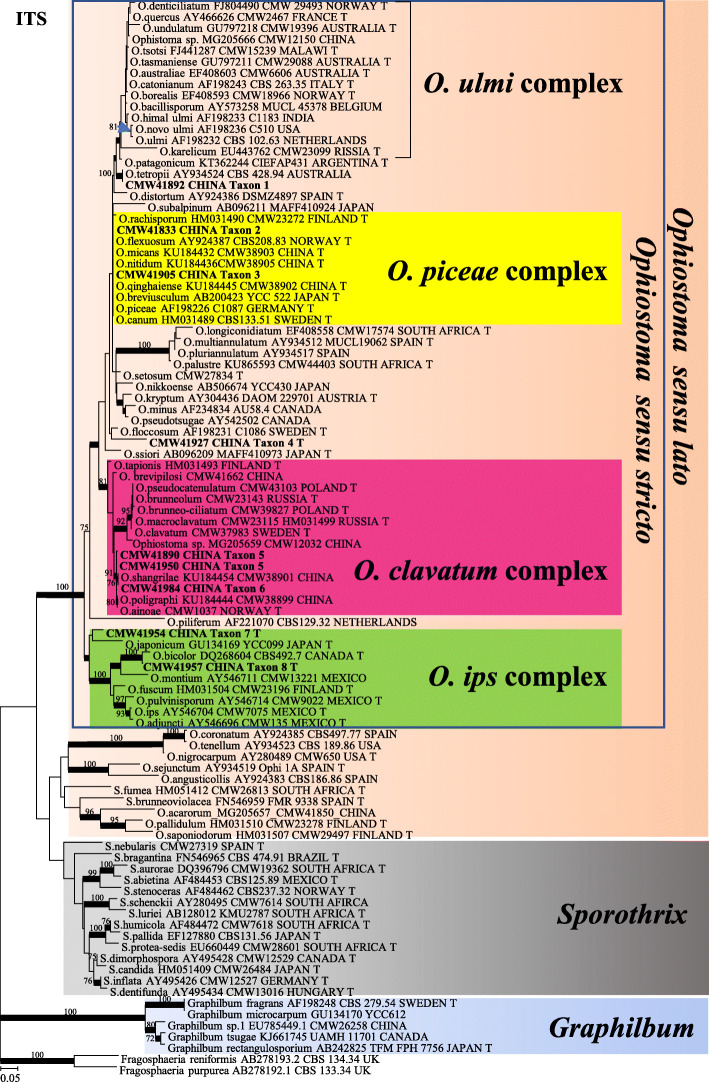
Phylogram obtained from ML analyses of the ITS region of *Ophiostoma*. Sequences obtained in this study are printed in bold type. ML bootstrap support values (1000 replicates, normal type) above 75% are indicated at the nodes. Posterior probabilities (above 0.9) obtained from BI are indicated by bold lines at the relevant branching points. T = ex-type cultures. Scale bar = total nucleotide difference between taxa

**Fig. 2:**
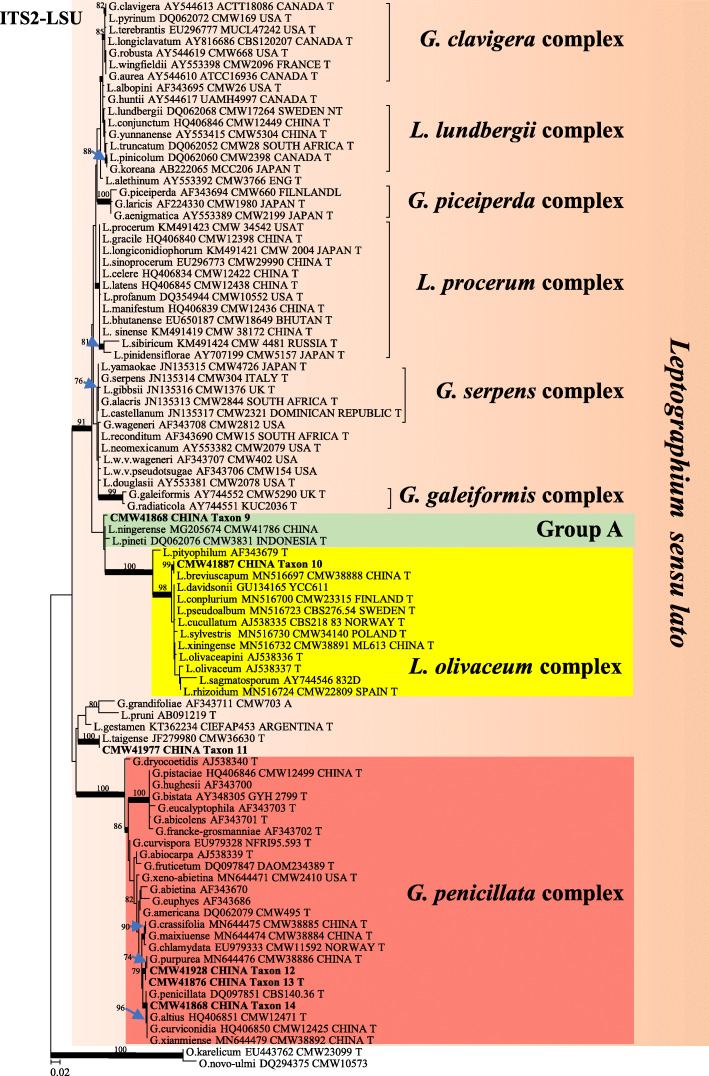
Phylogram obtained from ML analyses of the ITS2-LSU region of *Leptographium*. Sequences obtained in this study are printed in bold type. ML bootstrap support values (1000 replicates, normal type) above 75% are indicated at the nodes. Posterior probabilities (above 0.9) obtained from BI are indicated by bold lines at the relevant branching points. T = ex-type cultures. Scale bar = total nucleotide difference between taxa

Based on analysis of ITS and ITS-LSU sequence data, of the total 135 isolates collected in this study, 87 isolates resided in *Ophiostoma sensu stricto* (Fig. 1), and the remaining 48 isolates resided in *Leptographium s. lat.* (Fig. 2). Most of the isolates belonging to *Ophiostoma s. str.* resided in three species complexes namely the *O. piceae*-, *O. clavatum*- and *O. ips* species complexes, while most of the isolates belonging to *Leptographium s. lat.* resided in the *Grosmannia penicillata* species complex. Based on the availability of sequence data from previously studies, datasets of different protein coding gene regions were compiled and analysed separately for different species complexes. Phylogenetic analyses of these datasets separated the isolates into 14 distinct taxa (Table 2), 11 of which belonged to previously described species and three represented novel species.

**Table 2:** Isolates of ophiostomatoid fungi obtained from different mites in Qinghai. Species names of novel taxa are printed in bold type

Taxon	Species	Isolate number^**a,b**^		Host	Beetle	Mite^**c**^	Locations	GenBank number^**d**^		
		CMW	CBS					ITS/ITS2-LSU	***BT***	***EF***
1	*Ophiostoma tetropii*	41891		*Picea crassifolia*	*Dendroctonus micans*	M11	Maixiu	MH121623	MH124426	MH124490
		41892		*P. crassifolia*	*D. micans*	M11	Maixiu	MH121624	MH124427	MH124491
		41893		*P. crassifolia*	*D. micans*	M23	Maixiu	MH121625	MH124428	MH124492
		41938		*P. crassifolia*	*Ips nitidus*	M7	Xianmi	MH121626	MH124429	MH124493
2	*O. nitidum*	41883		*P. crassifolia*	*Polygraphus poligraphus*	M6	Maixiu	MH121627	MH124430	MH124494
		41874		*P. crassifolia*	*I. nitidus*	M9	Maixiu	MH121628	MH124431	MH124495
		41886		*P. crassifolia*	*P. poligraphus*	M6	Maixiu	MH121629	MH124432	MH124496
		41895		*P. crassifolia*	*D. micans*	M23	Maixiu	MH121630	MH124433	MH124497
		41898		*P. crassifolia*	*D. micans*	M3	Maixiu	MH121631	MH124434	MH124498
		41899		*P. crassifolia*	*D. micans*	M3	Maixiu	MH121632	MH124435	MH124499
		41901		*P. crassifolia*	*D. micans*	M23	Maixiu	MH121633	MH124436	MH124500
		41902		*P. crassifolia*	*D. micans*	M23	Maixiu	MH121634	MH124437	MH124501
		41911		*P. crassifolia*	*D. micans*	M23	Maixiu	MH121635	MH124438	MH124502
		41917		*P. crassifolia*	*D. micans*	M23	Maixiu	MH121636	MH124439	MH124503
		41918		*P. crassifolia*	*D. micans*	M23	Maixiu	MH121637	MH124440	MH124504
		41923		*Picea purpurea*	*Ips shangrila*	M6	Maixiu	MH121638	MH124441	MH124505
		41933		*P. purpurea*	*I. shangrila*	M17	Maixiu	MH121639	MH124442	MH124506
		41934		*P. purpurea*	*I. shangrila*	M17	Maixiu	MH121640	MH124443	MH124507
		41939		*P. crassifolia*	*I. nitidus*	M20	Xianmi	MH121641	MH124444	MH124508
3	*O. qinghaiense*	41900		*P. crassifolia*	*D. micans*	M3	Maixiu	MH121642	MH124445	MH124509
		41903		*P. crassifolia*	*D. micans*	M23	Maixiu	MH121643	MH124446	MH124510
		41905		*P. crassifolia*	*D. micans*	M23	Maixiu	MH121644	MH124447	MH124511
		41906		*P. crassifolia*	*D. micans*	M23	Maixiu	MH121645	MH124448	MH124512
		41907		*P. crassifolia*	*D. micans*	M23	Maixiu	MH121646	MH124449	MH124513
		41915		*P. crassifolia*	*D. micans*	M23	Maixiu	MH121647	MH124450	MH124514
4	***O. kunlunense***	41927	141903^H^	*P. purpurea*	*I. shangrila*	M19	Maixiu	MH121648	MH124451	MH124515
		48853	141904	*P. purpurea*	*I. shangrila*	M6	Maixiu	MH121649	MH124452	MH124516
		48854	141905	*P. purpurea*	*I. shangrila*	M18	Maixiu	MH121650	MH124453	–
5	*O. ainoae*	41882		*P. crassifolia*	*P. poligraphus*	M20	Maixiu	MH121651	MH124454	MH124517
		41890		*P. crassifolia*	*P. poligraphus*	M14	Maixiu	MH121652	MH124455	MH124518
		41958		*P. crassifolia*	*I. nitidus*	M18	Xianmi	MH121653	MH124456	MH124519
		41881		*P. crassifolia*	*P. poligraphus*	M20	Maixiu	MH121654	MH124457	MH124520
		41950		*P. crassifolia*	*I. nitidus*	M10	Xianmi	MH121655	MH124458	MH124521
6	*O. shangrilae*	41885		*P. crassifolia*	*P. poligraphus*	M6	Maixiu	MH121656	MH124459	MH124522
		41930		*P. purpurea*	*I. shangrila*	M18	Maixiu	MH121657	MH124460	MH124523
		41968		*P. crassifolia*	*I. nitidus*	M24	Xianmi	MH121658	MH124461	MH124524
		41983		*P. crassifolia*	*I. nitidus*	M5	Xianmi	MH121659	MH124462	MH124525
		41984		*P. crassifolia*	*I. nitidus*	M5	Xianmi	MH121660	MH124463	MH124526
7	***O. manchongi***	41872		*P. crassifolia*	*I. nitidus*	M10	Maixiu	MH121661	MH124464	MH124527
		41954	141906^H^	*P. crassifolia*	*I. nitidus*	M18	Xianmi	MH121662	MH124465	–
		41975	141907	*P. crassifolia*	*I. nitidus*	M5	Xianmi	MH121663	MH124466	MH124528
		41978		*P. crassifolia*	*I. nitidus*	M5	Xianmi	MH121664	MH124467	MH124529
		41979	141908	*P. crassifolia*	*I. nitidus*	M5	Xianmi	MH121665	MH124468	–
8	*O. bicolor*	41861		*P. crassifolia*	*I. nitidus*	–	Maixiu	MH121666	MH124469	–
		41877		*P. crassifolia*	*I. nitidus*	M15	Maixiu	MH121667	MH124470	MH124530
		41878		*P. crassifolia*	*P. poligraphus*	M20	Maixiu	MH121668	MH124471	MH124531
		41949		*P. crassifolia*	*I. nitidus*	M18	Xianmi	MH121669	MH124472	MH124532
		41957		*P. crassifolia*	*I. nitidus*	M18	Xianmi	MH121670	MH124473	MH124533
		41965		*P. crassifolia*	*I. nitidus*	M20	Xianmi	MH121671	MH124474	MH124534
9	*Leptographium ningerense*	41868		*P. crassifolia*	*I. nitidus*	M6	Maixiu	MH121672	MH124475	MH124535
10	*L. breviuscapum*	41887		*P. crassifolia*	*P. poligraphus*	M6	Maixiu	MH121673	MH124476	MH124536
11	*L. taigense*	41864		*P. crassifolia*	*I. nitidus*	–	Xianmi	MH121674	MH124477	MH124537
		41970		*P. crassifolia*	*I. nitidus*	M2	Xianmi	MH121675	MH124478	MH124538
		41977		*P. crassifolia*	*I. nitidus*	M5	Xianmi	MH121676	MH124479	MH124539
12	*Grosmannia purpurea*	41928		*P. purpurea*	*I. shangrila*	M18	Maixiu	MH121677	MH124480	MH124540
		41922		*P. purpurea*	*I. shangrila*	M18	Maixiu	MH121678	MH124481	MH124541
13	***G. zekuensis***	41862		*P. crassifolia*	*I. nitidus*	–	Maixiu	MH121679	MH124482	MH124542
		41870	141900	*P. crassifolia*	*I. nitidus*	M2	Maixiu	MH121680	–	MH124543
		41871		*P. crassifolia*	*I. nitidus*	M6	Maixiu	MH121681	MH124483	MH124544
		41875		*P. crassifolia*	*I. nitidus*	M9	Maixiu	MH121682	MH124484	MH124545
		41876	141901^H^	*P. crassifolia*	*I. nitidus*	M16	Maixiu	MH121683	MH124485	MH124546
		48852	141902	*P. crassifolia*	*I. nitidus*	M6	Maixiu	MH121684	MH124486	MH124547
14	*G. xianmiense*	41866		*P. crassifolia*	*I. nitidus*	M13	Maixiu	MH121685	MH124487	MH124548
		41867		*P. crassifolia*	*I. nitidus*	M18	Maixiu	MH121686	MH124488	MH124549
		41880		*P. crassifolia*	*P. poligraphus*	M20	Maixiu	MH121687	MH124489	MH124550

^a^ The culture collection (CBS) of Westerdijk Fungal Biodiversity Institute, Utrecht, the Netherlands; CMW Culture Collection of the Forestry and Agricultural Biotechnology Institute (FABI), University of Pretoria, Pretoria, South Africa

^b^ Hex-holotype isolate

^c^ Mite species, see Table 1

^d^*ITS* Internal transcribed spacer regions 1 and 2 of the nuclear ribosomal DNA operon, including the 5.8S region, *ITS2-LSU* The internal transcribed spacer 2 region and partial large subunit of the nrDNA operon, *ΒT* Beta-tubulin, *EF* Translation elongation factor 1-alpha

**Fig. 3:**
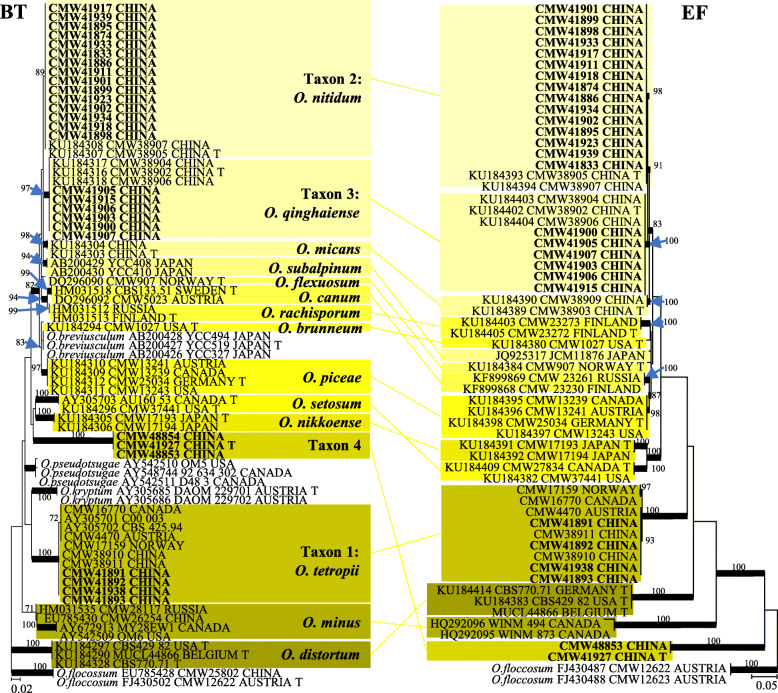
Phylogram obtained from ML analyses of the partial *BT* and *EF* gene of *O. piceae* complex. Sequences obtained in this study are printed in bold type. ML bootstrap support values (1000 replicates, normal type) above 75% are indicated at the nodes. Posterior probabilities (above 0.9) obtained from BI are indicated by bold lines at the relevant branching points. T = ex-type cultures. Scale bar = total nucleotide difference between taxa

**Fig. 4:**
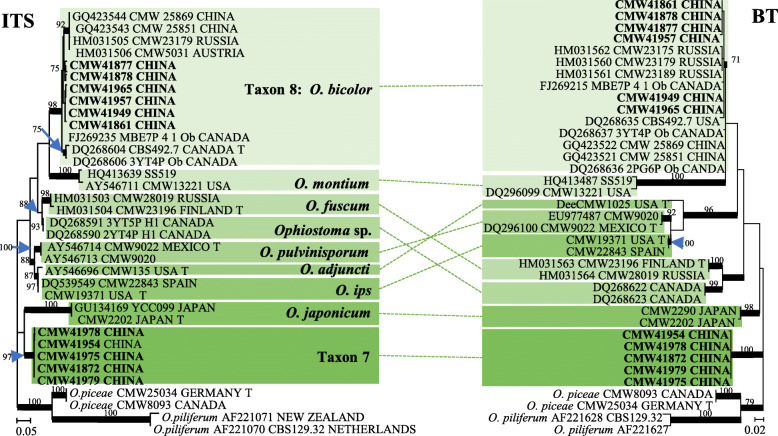
Phylogram obtained from ML analyses of the ITS region and the partial *BT* gene of *O. ips* complex. Sequences obtained in this study are printed in bold type. ML bootstrap support values (1000 replicates, normal type) above 75% are indicated at the nodes. Posterior probabilities (above 0.9) obtained from BI are indicated by bold lines at the relevant branching points. T = ex-type cultures. Scale bar = total nucleotide difference between taxa

In *Ophiostoma s. str.*, two taxa (Taxa 1 and 4) represented by four and three isolates respectively, grouped peripheral to the *O. piceae* complex in the ITS tree (Fig. 1). Analyses of *BT* and *EF* sequences data (Fig. 3) showed that Taxon 1 belong to *O. tetropii*, and isolates of Taxon 4 formed a distinct, well supported clade (with 100% ML bootstrap support and BI posterior probabilities larger than 0.9 for both *BT* and *EF* gene regions) which was separated from all previously described species, and thus this taxon represented a novel species. Taxa 2 and 3, represented by 32 and 11 isolates respectively, grouped with *O. nitidus* and *O. qinghaiense* in *O. piceae* complex. Taxa 5 and 6 grouped in the *O. clavatum* complex (Fig. 1) and were represented by ten and 11 isolates respectively. Based on the *BT* and *EF* sequence data (Fig. S1) the Taxon 5 isolates grouped in a single clade with the ex-type isolates of both *O. ainoae* and *O. poligraphi*, suggesting that these represented a single species. Taxon 6 isolates grouped with sequences of *O. shangrilae*. Taxon 7 resided close to and Taxon 8 within the *O. ips* complex (Fig. 1) and were represented by ten and six isolates respectively. Based on ITS and *BT* data (Fig. 4) isolates of Taxon 7 formed a distinct and well supported clade (with 97% ML bootstrap support for ITS, 100% ML bootstrap support for *BT*, and BI posterior probabilities larger than 0.9 for both ITS and *BT* gene regions) which was closest to, but clearly distinct from *O. japonicum,* and thus this taxon represented a novel species. Taxon 8 isolates grouped in a monophyletic lineage with several isolates of *O. bicolor*.

**Fig. 5:**
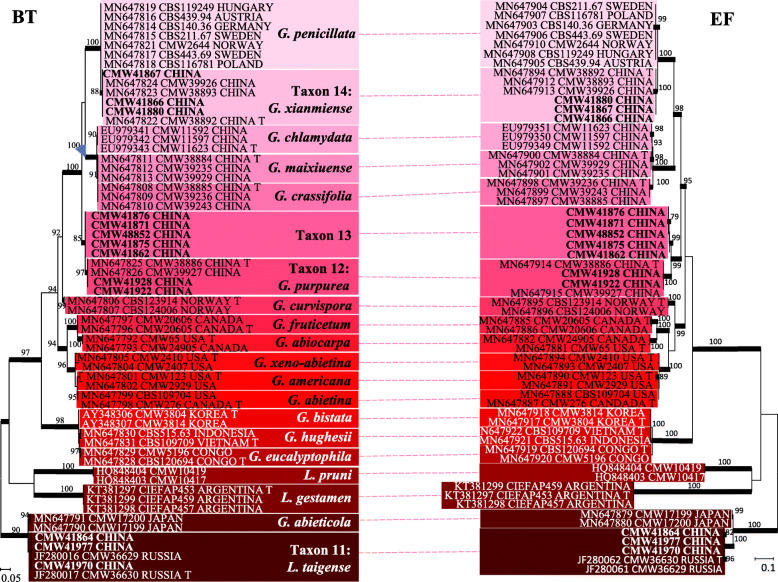
Phylogram obtained from ML analyses of the partial *BT* and *EF* gene of *G. penicillata* complex. Sequences obtained in this study are printed in bold type. ML bootstrap support values (1000 replicates, normal type) above 75% are indicated at the nodes. Posterior probabilities (above 0.9) obtained from BI are indicated by bold lines at the relevant branching points. T = ex-type cultures. Scale bar = total nucleotide difference between taxa

In *Leptographium s. lat.*, Taxon 9 represented by only one isolate grouped in Group A of *Leptographium s. lat.* together with *L. pineti* and *L. ningerensis* (Fig. 2) and *BT* and *EF* sequence analyses confirmed this Taxon was conspecific with *L. ningerensis* (Fig. S2). Taxon 10 grouped in the *L. olivaceum* complex (Fig. 2) and *BT* and *EF* sequence analyses confirmed that this isolate represented *L. breviuscapum* (Fig. S2). Taxon 11 grouped peripheral to the *G. penicillata* complex with *L. taigense* (Fig. 2) and *BT* and *EF* sequences analyses confirmed the identity of the species as *L. taigense* (Fig. 5). Taxa 12, 13, and 14 grouped in the *G. penicillata* complex (Fig. 2) and based on *BT* and *EF* sequences (Fig. 5), Taxon 12 was identified as *G. purpurea*, and Taxon 14 was identified as *G. xianmiense,* while isolates of Taxon 13 formed a distinct and well supported clade (with ML bootstrap supports larger than 95%, and BI posterior probabilities larger than 0.9 for both *BT* anf *EF* gene regions), which was close to but different from *G. purpurea*, thus this taxon represented a novel species.

### Frequencies of isolation

The most frequently collected mite species were *Uropodoidea* sp. 4 and *Uropodoidea* sp. 6 each of which represented 21.2% of all mites (Table 1). The next most frequently collected species was *Insectolaelaps* sp. 2 which represented 13.9% of the mites. A few species were collected at very low frequencies, such as *Uropodoidea* sp. 7 and *Uropodoidea* sp. 8 that only represented 0.6% of the collections.

The most frequently isolated ophiostomatoid fungi were *O. nitidum* (Taxon 2) and *L. taigense* (Taxon 11), which represented 23.5 and 22.8% of the total isolates respectively (Table S3). This was followed by *O. qinghaiense* (Taxon 3, 8.1%) and *O. shangrilae* (Taxon 6, 8.1%), and *O. ainoae* (Taxon 5, 7.4%) and Taxon 8 (7.4%). The fungi with the lowest frequency of isolations were *L. breviuscapum* (Taxon 10) and *L. ningerensis* (Taxon 9), both of which represented 0.7% of total isolates. The remaining fungi were found at frequencies lower than 5%.

The number of fungal isolates collected from different mite species differed substantially (Table S3). About 20% of the fungi were isolated from mites in the family *Winterschmidtiidae*. Twenty-four isolates collected from *Uropodoidea* sp. 4 represented 17% of the total isolates. This was followed by 12.1% of the isolates collected from *Insectolaelaps* sp. 2, 10% isolates collected from *Insectolaelaps* sp. 1, 10.6% isolates collected from *Uropodoidea* sp. 6 (Table S3).

The 33 isolates collected from the mites in *D. micans* galleries represented 23.4% of the total number of isolates, while 75 isolates from the mites in *I. nitidus* galleries represented 53.2%, 19 isolates from the mites in *I. shangrila* galleries represented 14.2%, and the remaining 13 isolates from mites in *P. poligraphus* galleries represented 9.2% of the isolates.

## TAXONOMY

Based on the phylogenetic analyses of different gene regions, as discussed above, three out of the total 14 taxa identified in this study represented novel species, and descriptions for these novel species are provided below. Phylogenetic analyses of *BT* and *EF* gene regions (Fig. S1) also indicated that *O. ainoae* and *O. poligraphi* belonged to the same species, thus *Ophiostoma poligraphi* is synonymized with *Ophiostoma ainoae*.

### Taxon 4

**Ophiostoma kunlunense** R.L. Chang & Z.W. de Beer, **sp. nov.**

MycoBank MB 827335

(Fig. 6)

**Fig. 6:**
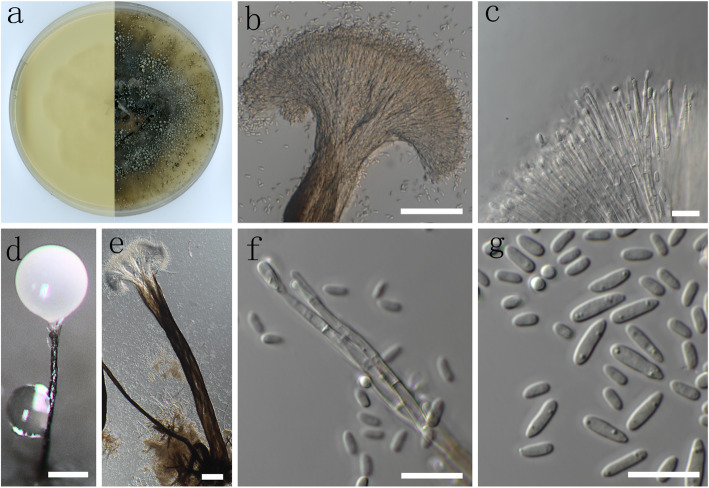
Morphological characters of asexual structure of *Ophiostoma kunlunense* sp. nov. **a** Cultures on malt extract agar (MEA); **b**, **d**, **e** Pesotum asexual state; **c**, **f** conidiogenous cells; **g** conidia. –Scale bars: **b** = 50 μm; **c**, **f**, **g** = 10 μm; **d** = 200 μm; **e** = 100 μm

*Etymology*. Name refers to the Kunlun mountains, which is one of the longest mountain ranges in Asia, that ends in Qinghai province from where this fungus was first isolated.

*Diagnosis*: *Ophiostoma kunlunense* is phylogenetically distinct from all other species in the *O. piceae* complex, and groups closest to species slightly peripheral to the complex like *O. floccosum, O. setosum* and *O. nikkoense*. It produces a pesotum-like asexual morph similar to other species in the complex, and species peripheral to the complex such as *O. nikkoense* and *O. setosum*. However, both *O. kunlunense* and *O. nikkoense* lack the sporothrix-like synasexual state that characterizes *O. floccosum, O. setosum* and most other species in the complex. In addition, *O. floccosum* is distinguished by yellow conidial masses, while *O. kunlunense* and all the other species produce white conidial masses (Harrington et al. 2001). *Ophiostoma nikkoense* is distinguished from *O. kunlunense* and the other species by its extremely elongated, clavate and septate conidia (Yamaoka et al. 2004).

*Type*: **China**: *Qinghai province*: Maixiu Forest Farm, from *Uropodoidea* sp. in gallery of *Ips shangrila* on *Picea purpurea*, 8 Aug. 2010, *S. J. Taerum * (PREM61583 – holotype (dried culture); CMW41927 = CBS141903 – ex-type culture).

*Description*: *Sexual morph* not observed. *Asexual morph* pesotum-like, occurring singly or in groups of up to 15, macronematous, synnematous, erect, (511–) 705–1729.5 (− 1301) μm long, including condiogenous apparatus. *Conidia* hyaline, 1-celled, smooth, oblong, clavate or obovoid (3.5–) 3–6 (− 8) × 1.5–2(− 2.5) μm, accumulating in a white, gelatinous mass at the apex of the synnema.

*Culture characteristics*: *Colonies* hyaline or dark brown when synnemata form. Mycelium superficial on the agar. Pesotum-like asexual morph dominant in the cultures. Optimal temperature for growth 20 °C, reaching 56.6 mm diam in 10 d. No growth observed at 5 °C or 30 °C and above.

*Additional specimens examined*: China: *Qinghai province*: Maixiu Forest Farm, from *Insectolaelaps* sp. in gallery of *Ips shangrila* on *Picea purpurea*, 8 Aug. 2010, *S. J. Taerum* (PREM61584–dried culture; CMW48853 = CBS141904 – culture); ibid., 8 Aug. 2010, *S. J. Taerum*(PREM61585 – dried culture; CMW48854 = CBS141905 – culture).

### Taxon 5

**Ophiostoma ainoae** H. Solheim, *Nord. J. Bot*. **6**: 201 (1986).

*Synonym*: *Ophiostoma poligraphi* M.L. Yin et al. *Fungal Biol*. **120**: 464 (2016).

*Notes*: When *BT* and *EF* sequences produced for several isolates obtained in the present study were analysed together with sequences of three *O. ainoae* isolates from the study of Linnakoski et al. (2016a) and two *O. poligraphi* isolates from the study of Yin et al. (2016), it became clear that the latter two groups of isolates represented geographically isolated populations of the same species. Sequences of our isolates did not group consistently with isolates in either of the two clades (Fig. S1). For the *BT* region, our isolates grouped between the two ‘species’, while in the *EF* analyses, they all grouped with *O. poligraphi*. There are confirmed reports of *O. ainoae* from *Ips typographus* and *Pityogenes chalcographus* on *Picea abies* in Europe (Linnakoski et al. 2016a), while *O. poligraphi* was described from *Polygraphus poligraphus* and *Dendroctonus micans* on *Pi. crassifolia* in Qinghai (Yin et al. 2016). Our isolates were from mites on *P. poligraphus* and *Ips nitidus,* also from *Pi. crassifolia* in Qinghai, and this is consistent with the host and beetle vectors of both species. Although a sexual state has not been described for *O. poligraphi*, the broad synnemata of this species also correspond with those described for *O. ainoae* (Solheim 1986), supporting the synonymy of the two species.

### Taxon 7

**Ophiostoma manchongi** R.L. Chang & Z.W. de Beer, **sp. nov.**

MycoBank MB 827336

(Fig. 7)

**Fig. 7:**
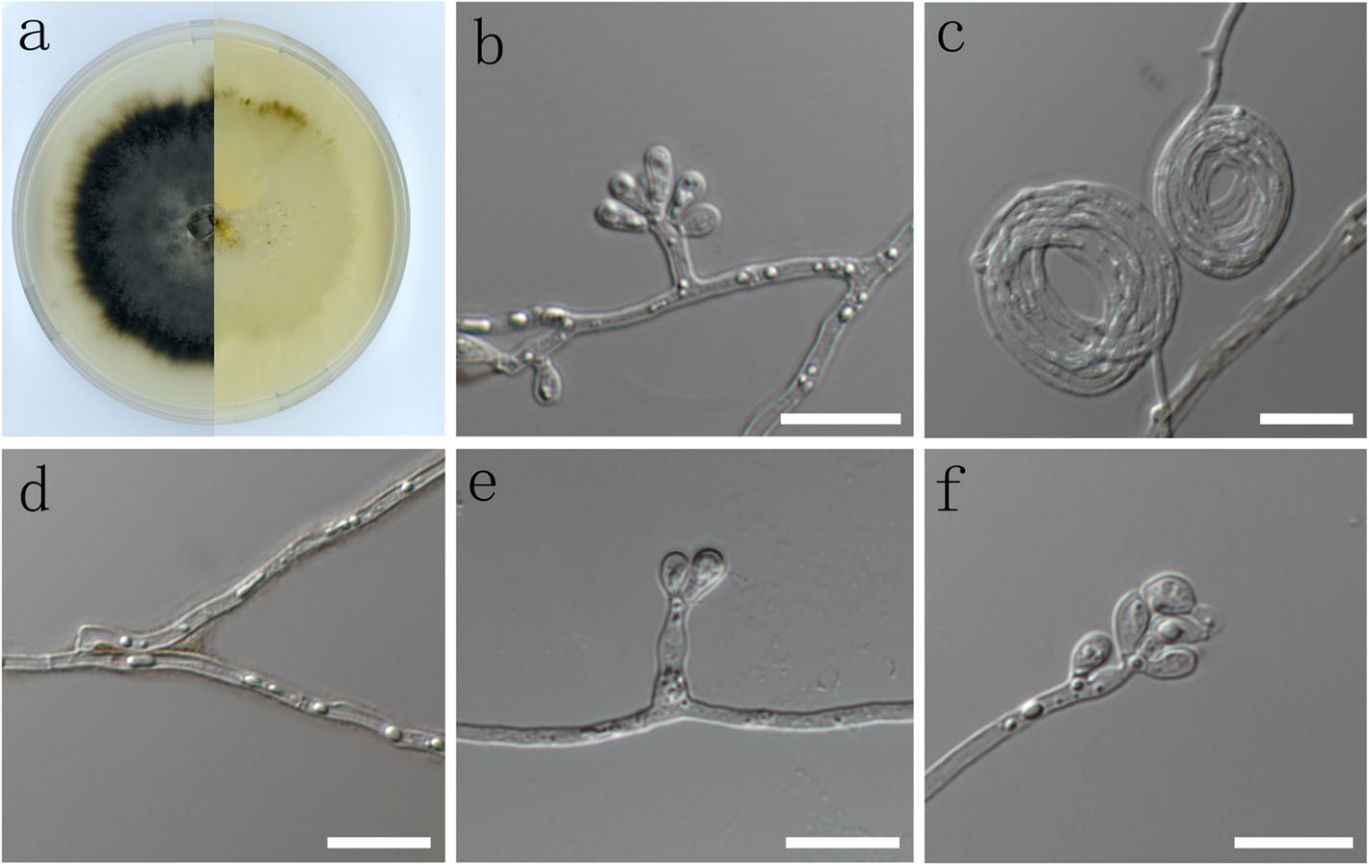
Morphological characters of asexual structure of *Ophiostoma manchongi* sp. nov. **a** Cultures on malt extract agar (MEA); **b**, **e**, **f** conidiogenous cells and conidia; **c**, **d** mycelium. –Scale bars: 10 μm

*Etymology*. Name refers to *manchong*, the Chinese word for mite.

*Diagnosis*: Both ITS and *BT* sequences clearly distinguish between *O. manchongi* and its closest relative, *O. japonicum*. In addition, the sporothrix-like asexual morph of *O. manchongi,* producing obovoid conidia, is very different from the light coloured synnematous asexual state of *O. japonicum* with its cylindrical or clavate conidia (Yamaoka et al. 1997).

*Type*: **China**: *Qinghai province*: Xianmi Forest Farm, from *Uropodoidea* sp. in gallery of *Ips shangrila* on *Picea purpurea*, 8 Aug. 2010, *S. J. Taerum * (PREM61580 – holotype (dried culture); CMW41954 = CBS141906 – ex-type culture).

*Description*: *Sexual morph* not observed. *Asexual morph* sporothrix-like, erect, arising directly from the mycelium. *Conidia* hyaline, 1-celled, smooth, oblong, obovoid (3–) 4–5.5 (− 7) x (1.5–) 2–3 (− 4.5) μm.

*Culture characteristics*: colonies at first hyaline, later becoming dark brown at the centre. Mycelium superficial on the agar. Optimal temperature for growth 25 °C, reaching 29.6 mm diam in 10 d. No growth at 5 °C or 35 °C.

*Specimens examined*: **China**: *Qinghai province*: Xianmi Forest Farm, from *Insectolaelaps* sp. in gallery of *Ips nitidus* on *Picea crassifolia*, 8 Aug. 2010, *S. J. Taerum* (PREM61581–dried culture; CMW42975 = CBS141907 culture); ibid., from *Uropodoidea* sp. in gallery of *Ips shangrila* on *Picea purpurea*, 8 Aug. 2010, *S. J. Taerum* (PREM61582 – dried culture; CMW41979 = CBS141908 – culture).

### Taxon 13

**Grosmannia zekuensis** R.L. Chang & Z.W. de Beer, **sp. nov.**

MycoBank MB 827337

(Fig. 8)

**Fig. 8:**
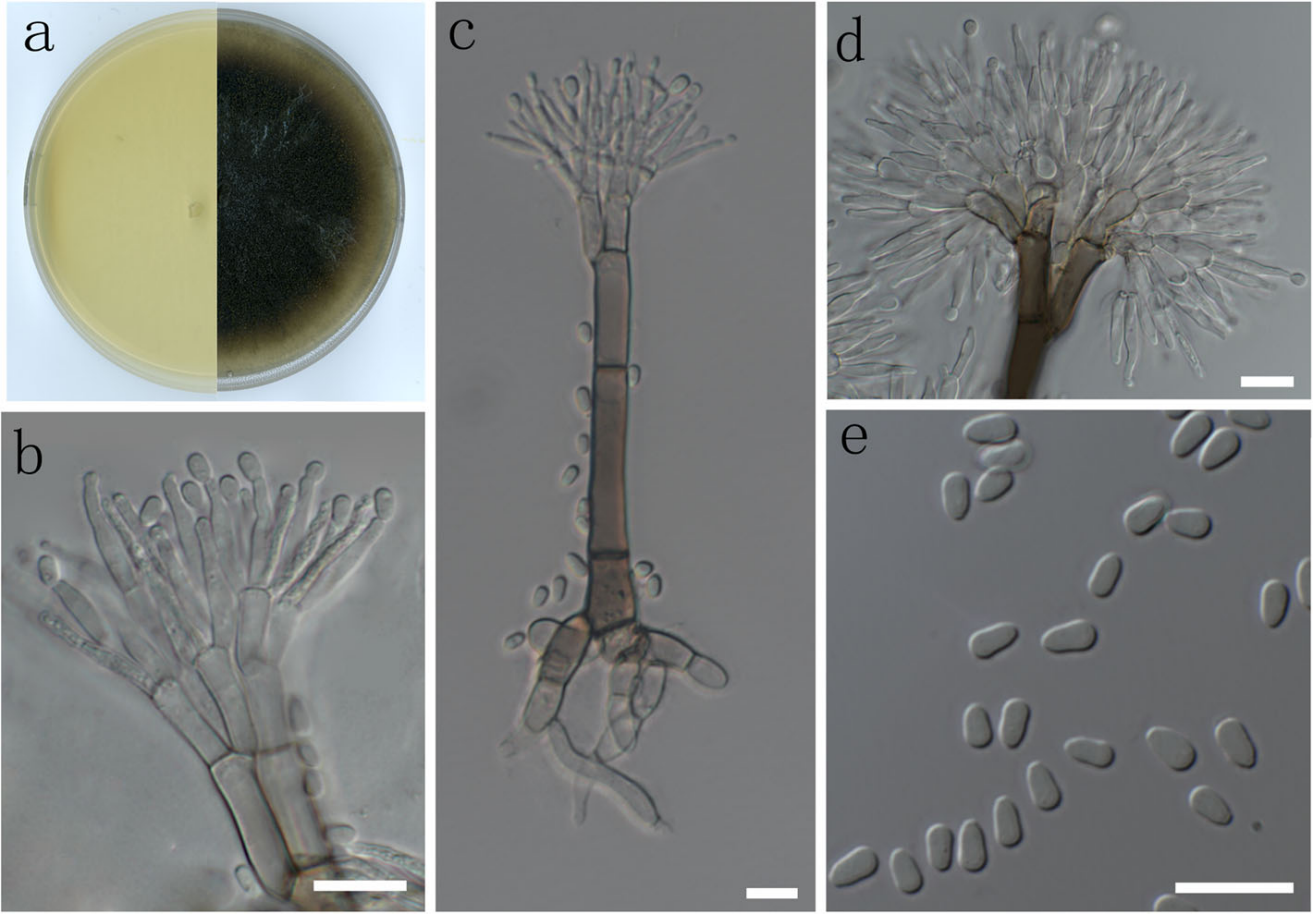
Morphological characters of asexual structure of *Grosmannia zekuensis* sp. nov. **a** Cultures on malt extract agar (MEA); **b**, **d** conidiogenous apparatus; **c** conidiophore; **e** conidia. Scale bars: 10 μm

*Etymology*: After Zeku, the county where samples were collected.

*Diagnosis*: *Grosmannia zekuensis* is closely related to *G. purpurea* based on *BT* and *EF* sequences*,* but the two species can be readily distinguished based on morphology and growth rate. *G. zekuensis* forms smaller asexual structures and conidia than *G. purpurea*, which is evident when the lengths of the following structures are compared: *stipes* 67–269 vs 100–170 μm; *conidiogenous apparatus* 16–67 vs 177–162 μm; *conidiogenous cells* 6–15 vs 70–110 μm; *conidia* 3.5–8 vs 10–15 μm. However, *G. zekuensis* grows between 46 and 73 mm diam on 2% MEA in 8 d at 25 °C (varying between different isolates), while *G. purpurea* isolates grow on average around 32 mm diam under the same conditions (Yin et al. 2020).

*Type*: **China**: *Qinghai province*: Xianmi Forest Farm, from *Bakerdania* sp. in gallery of *Ips nitidus* on *Picea crassifolia*, 8 Aug. 2010, *S. J. Taerum* (PREM61579 – holotype (dried culture); CMW41876 = CBS141901 – ex-type culture).

*Description*: *Sexual morph* not observed. *Conidiophores* macronematous, mononematous, erect, arising directly from the mycelium, (98–) 114–182 (− 269) μm long. *Rhizoids* present. *Stipes* olivaceous, 2–4 septate, not constricted at septa, (67–) 73.5–125.5 (− 182) μm long; apical cells occasionally swollen at apex, (4–) 5–7.5 (− 10.5) μm wide; basal cells not swollen, (5.5–) 7–10 (− 11) μm wide. *Conidiogenous apparatus* (16–) 30.5–49 (− 67) μm long, excluding the conidial mass, with multiple series of cylindrical branches; primary branches olivaceous, smooth, cylindrical, not swollen at apex, aseptate, arrangement of primary branches was Type B—more than two branches, (7.5–) 10–16 (− 16) x (3–) 3.5–4.5 (− 5.5) μm; secondary branches light olivaceous, frequently swollen at apex, aseptate, (6–) 8–11 (− 12.5) x (2–) 2.5–4 (− 5) μm; tertiary branches light olivaceous, aseptate, (6–) 7–10 (− 11) x (2–) 2.5–3 (− 3) μm. *Conidiogenous cells* discrete, hyaline, 2–3 per branch, aseptate, cylindrical, tapering slightly at the apex, (6–) 8–12 (− 14.5) x (1.5–) 2–3 (− 3) μm. *Conidia* hyaline, aseptate, elliptical, (3.5–) 4–5.5 (− 8) x (2–) 2.5–3 (− 3.5) μm.

*Culture characteristics*: *Colonies* hyaline without asexual structures or dark brown when asexual structures form. Mycelium superficial on the agar. Optimal temperature for growth 25 °C. Some isolates grow faster, reaching 73.3 mm diam in 8 d at 25 °C and no growth at 30 °C and above. However, some isolates grow slowly and only reach 46.3 mm diam in 8 d and slow growth observed at 35 °C.

*Additional specimens examined*: **China**: *Qinghai province*: Xianmi Forest Farm, from *Insectolaelaps* sp. in gallery of *Ips nitidus* on *Picea crassifolia*, 8 Aug. 2010, *S. J. Taerum* (PREM61578 – dried culture; CMW48852 = CBS141902 – culture); ibid., from *Schwiebea wainsteini* in gallery of *Ips nitidus* on *Picea crassifolia*, 8 Aug. 2010, *S. J. Taerum* (PREM61577 – dried culture; CMW41870 = CBS141900 – culture).

## DISCUSSION

We collected 173 mites representing 18 species from spruce-infesting bark beetles in Qinghai province, China. *Uropodoidea* sp. 4 and *Uropodoidea* sp. 6, were most abundant among the mites. We obtained 135 fungal isolates from 65 mite individuals. Based on DNA sequence data, 14 fungal species were identified, eight species of *Ophiostoma* and six of *Leptographium s. lat*. Among these species, three were recognized as novel taxa and were thus described and provided with names. Of the remaining 11 species, ten had previously been reported from China. *Ophiostoma tetropii* is reported for the first time from China.

The only previous study to report on ophiostomatoid fungi associated with phoretic mites in China was conducted on mite associates of six pine-infesting bark beetles in Yunnan (Chang et al. 2017). The species diversity of mites collected in the present study from Qinghai was higher than that found in Yunnan (18 species vs. 13), despite the fact that only four beetle species were sampled in Qinghai as opposed to six in Yunnan. However, more mite individuals (173) were collected in Qinghai from two host trees than the 106 mites from only *Pinus kesiya* in Yunnan (Chang et al. 2017). Only *Insectolaelaps* sp. 1 was collected from both Qinghai and Yunnan. This species was the most frequently collected species in Yunnan and the fourth most frequently collected species in Qinghai, suggesting that it is widely distributed on conifer hosts in western China. The fact that only one species was shared between the two regions is perhaps not surprising because the mites were collected from different bark beetle species, different tree species, and different climatic zones.

Together with the previous report from Yunnan (Chang et al. 2017), a total of 31 species of mites associated with ten bark beetles have now been reported from western China. This number is much less than 270 mite species that have been reported in association with more than 110 bark beetle species from the many studies in North America and Europe (Hofstetter et al. 2015), 12 mite species were reported from *Ips typographus* in Japan, of which only three species did not occur in Europe (Moser et al. 1997). Because most of the mites in our study have not been identified to species level, and because our sampling strategy was aimed at isolating fungi and not only the collection of mites, it is not possible to compare the presence or absence of mite species with other studies. However, at a higher taxonomic level, the most frequent mite order associated with bark beetles was the *Mesostigmata* in both China and collections collectively from Europe and North America, where it is represented by 20 and 140 species respectively (Hofstetter et al. 2015).

The fungal species diversity from mites in Qinghai was somewhat higher when compared with the diversity of fungal species from mites in Yunnan (Chang et al. 2017), with 12 species reported from Yunnan versus 14 species in Qinghai, although the total number of fungal isolates was smaller in Qinghai. The fungi collected in Qinghai all belonged to only two genera in the *Ophiostomatales*, *Ophiostoma* and *Leptographium s. lat.*, while isolates from Yunnan belonged to five genera, four (*Graphilbum*, *Leptographium s. lat.*, *Ophiostoma* and *Sporothrix*) in the *Ophiostomatales*, and *Graphium* in the *Microascales*. The fungal community collected from bark beetle-associate mites in Qinghai was very different to that in Yunnan. Only one fungal species, *L. ningerensis*, was shared between Qinghai and Yunnan.

A comparison of the fungal species from spruce infesting bark beetles in Qinghai (Yin et al. 2016) with those from mites in our study, revealed that eight species were found in both studies. These included: *L. breviuscapum*, *G. purpurea*, *G. xianmiense*, *O. nitidum*, *O. qinghaiense*, *O. ainoae*, *O. shangrilae* and *O. tetropii*. Not surprisingly, a large number of fungal species were also shared between galleries and mites in Yunnan province (Chang et al. 2017).

Apart from the three new species discovered in this study, we also collected and identified *O. tetropii* for the first time from China. This fungus was first described from spruce tree attacked by *Tetropium* sp. in Sweden (Mathiesen 1951), and was subsequently also reported from *Picea abies* attacked by *Ips typographus* and *Pityogenes chalcographus* in Finland (Linnakoski et al. 2010) and *Picea rubens* attacked by *Tetropium fuscum* in Canada (Harrison and Smith 2013). Its presence on mites in China is surprising because it was considered as an associate and indicator of beetles such as *T. fuscum* (Harrison and Smith 2013).

## CONCLUSIONS

The results of this study conducted in China reaffirmed the findings elsewhere in the world showing that there are close associations between bark beetle-associated mites and ophiostomatoid fungi. The sampling area, beetle species and hosts considered was relatively limited. Yet many undescribed species of mites and various novel taxa in *Ophiostoma* and *Leptographium* emerged from the study. This reflects how little is known regarding the ecology of bark beetles and their symbionts. This is not only in China but also in many other parts of the world. Although relatively high numbers of mites and fungal species were found in in this study, the survey covered a relatively small geographical area. Larger surveys with more systematic sampling are needed in the future to elucidate the ecological roles and specificity of fungus-vector relationships. However, what is clear is that mites may act as important vectors of fungal species other than those vectored by the bark beetles. These mites and their fungi should therefore also be considered in pest risk assessments which often only focus on the bark beetles and their fungi.

## Supplementary information

**Additional file 1: Fig. S1.** Phylogram obtained from ML analyses of the partial *BT* and *EF* gene of *Ophiostoma clavatum* complex. Sequences obtained in this study are printed in bold type. ML and MP bootstrap support values (1000 replicates, normal type) above 75% are indicated at the nodes. Posterior probabilities (above 0.9) obtained from BI are indicated by bold lines at the relevant branching points. T = ex-type cultures. Scale bar = total nucleotide difference between taxa.

**Additional file 2: Fig. S2.** Phylogram obtained from ML analyses of the partial *BT* and *EF* gene of *Leptographium olivaceum* complex. Sequences obtained in this study are printed in bold type. ML bootstrap support values (1000 replicates, normal type) above 75% are indicated at the nodes. Posterior probabilities (above 0.9) obtained from BI are indicated by bold lines at the relevant branching points. T = ex-type cultures. Scale bar = total nucleotide difference between taxa.

**Additional file 3: Table S1.** Ophiostomatoid fungi reported from China.

**Additional file 4: Table S2.** Numbers of mite individuals collected and numbers of mite individuals carring fungi in this study.

**Additional file 5: Table S3.** Numbers of fungal isolates associated with mites from different beetle galleries. Shades of red indicate number of fungal isolates.

## Data Availability

All data generated or analysed during this study are included in this published article [and its supplementary information files].

## Abbreviations

BI: Bayesian inference
*BT*: β-tubulin
*EF*: Elongation factor 1-α
FABI: Forestry and Agricultural Biotechnology Institute
ITS: The internal transcribed spacer regions 1 and 2
LSU: The nuclear large subunit
MEA: Malt extract agar
ML: Maximum likelihood
*s. lat.*: *sensu lato*
*s. str.*: *sensu stricto*
