# Supplementary material for: Ophiostomatoid fungi associated with mites phoretic on bark beetles in Qinghai, China
Source: IMA Fungus. 2020 Jul 30;11:15. doi: 10.1186/s43008-020-00037-9 (PMC7391587; doi:10.1186/s43008-020-00037-9)
Supplement: Supplementary file 1 — Additional file 1: Fig. S1. Phylogram obtained from ML analyses of the partial BT and EF gene of Ophiostoma clavatum complex. Sequences obtained in this study are printed in bold type. ML and MP bootstrap support values (1000 replicates, normal type) above 75% are indicated at the nodes. Posterior probabilities (above 0.9) obtained from BI are indicated by bold lines at the relevant branching points. T = ex-type cultures. Scale bar = total nucleotide difference between taxa. [file 43008_2020_37_MOESM1_ESM.pptx]

## Slide 1
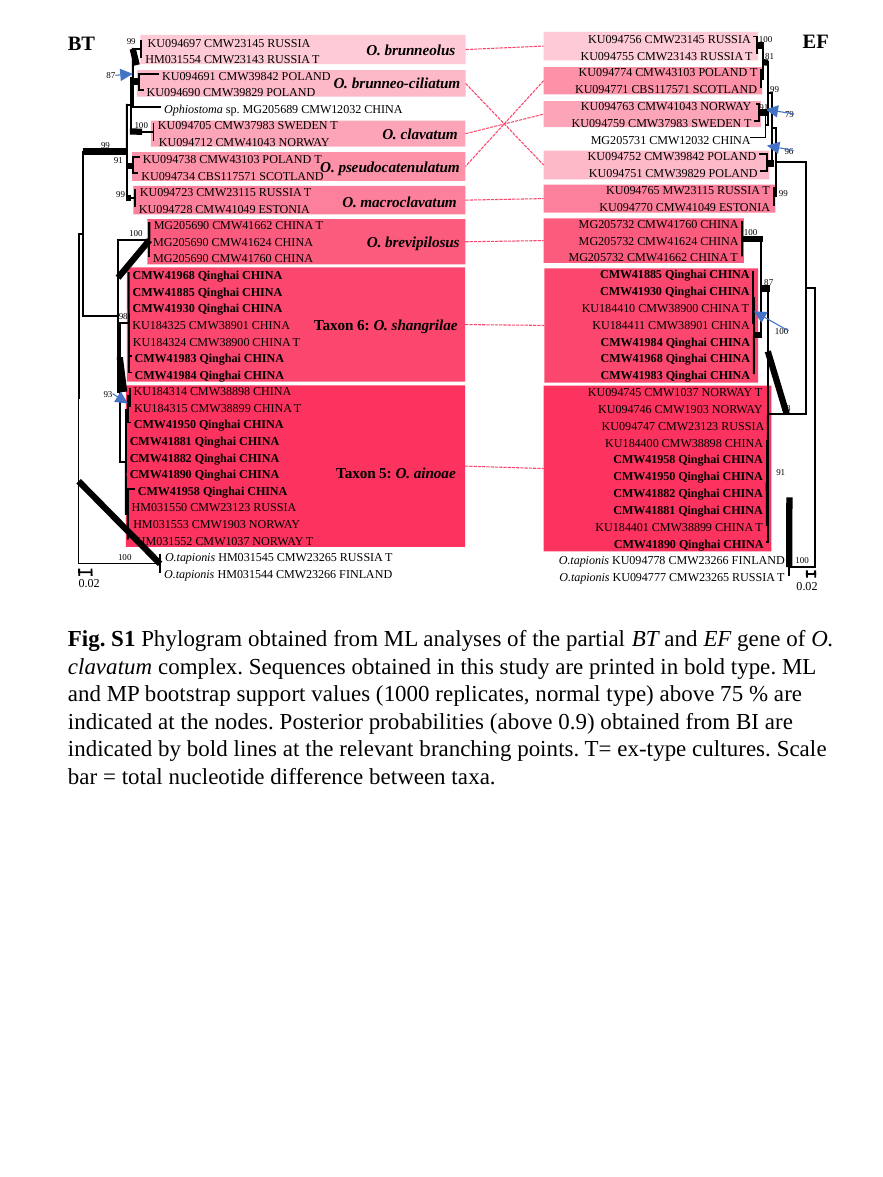

EF
BT
 KU094756 CMW23145 RUSSIA
100
 KU094755 CMW23143 RUSSIA T
81
 KU094774 CMW43103 POLAND T
 KU094771 CBS117571 SCOTLAND
99
 KU094763 CMW41043 NORWAY
91
79
 KU094759 CMW37983 SWEDEN T
MG205731 CMW12032 CHINA
96
 KU094752 CMW39842 POLAND
 KU094751 CMW39829 POLAND
KU094765 MW23115 RUSSIA T
99
KU094770 CMW41049 ESTONIA
MG205732 CMW41760 CHINA
100
 MG205732 CMW41624 CHINA
 MG205732 CMW41662 CHINA T
 CMW41885 Qinghai CHINA
87
 CMW41930 Qinghai CHINA
KU184410 CMW38900 CHINA T
 KU184411 CMW38901 CHINA
100
 CMW41984 Qinghai CHINA
 CMW41968 Qinghai CHINA
 CMW41983 Qinghai CHINA
KU094745 CMW1037 NORWAY T
KU094746 CMW1903 NORWAY
98
KU094747 CMW23123 RUSSIA
KU184400 CMW38898 CHINA
 CMW41958 Qinghai CHINA
91
 CMW41950 Qinghai CHINA
 CMW41882 Qinghai CHINA
 CMW41881 Qinghai CHINA
KU184401 CMW38899 CHINA T
 CMW41890 Qinghai CHINA
 O.tapionis KU094778 CMW23266 FINLAND
100
 O.tapionis KU094777 CMW23265 RUSSIA T
0.02
O. brunneolus
 KU094697 CMW23145 RUSSIA
99
 HM031554 CMW23143 RUSSIA T
 KU094691 CMW39842 POLAND
87
 KU094690 CMW39829 POLAND
 Ophiostoma sp. MG205689 CMW12032 CHINA
 KU094705 CMW37983 SWEDEN T
100
 KU094712 CMW41043 NORWAY
99
 KU094738 CMW43103 POLAND T
91
 KU094734 CBS117571 SCOTLAND
 KU094723 CMW23115 RUSSIA T
99
 KU094728 CMW41049 ESTONIA
 MG205690 CMW41662 CHINA T
100
 MG205690 CMW41624 CHINA
 MG205690 CMW41760 CHINA
 CMW41968 Qinghai CHINA
 CMW41885 Qinghai CHINA
 CMW41930 Qinghai CHINA
98
 KU184325 CMW38901 CHINA
 KU184324 CMW38900 CHINA T
 CMW41983 Qinghai CHINA
 CMW41984 Qinghai CHINA
 KU184314 CMW38898 CHINA
93
 KU184315 CMW38899 CHINA T
 CMW41950 Qinghai CHINA
 CMW41881 Qinghai CHINA
 CMW41882 Qinghai CHINA
 CMW41890 Qinghai CHINA
 CMW41958 Qinghai CHINA
 HM031550 CMW23123 RUSSIA
 HM031553 CMW1903 NORWAY
 HM031552 CMW1037 NORWAY T
 O.tapionis HM031545 CMW23265 RUSSIA T
100
 O.tapionis HM031544 CMW23266 FINLAND
0.02
O. brunneo-ciliatum
O. clavatum
O. pseudocatenulatum
 O. macroclavatum
 O. brevipilosus
 Taxon 6: O. shangrilae
Taxon 5: O. ainoae
Fig. S1 Phylogram obtained from ML analyses of the partial BT and EF gene of O. clavatum complex. Sequences obtained in this study are printed in bold type. ML and MP bootstrap support values (1000 replicates, normal type) above 75 % are indicated at the nodes. Posterior probabilities (above 0.9) obtained from BI are indicated by bold lines at the relevant branching points. T= ex-type cultures. Scale bar = total nucleotide difference between taxa.
