# Supplementary material for: Ophiostomatoid fungi associated with mites phoretic on bark beetles in Qinghai, China
Source: IMA Fungus. 2020 Jul 30;11:15. doi: 10.1186/s43008-020-00037-9 (PMC7391587; doi:10.1186/s43008-020-00037-9)
Supplement: Supplementary file 2 — Additional file 2: Fig. S2. Phylogram obtained from ML analyses of the partial BT and EF gene of Leptographium olivaceum complex. Sequences obtained in this study are printed in bold type. ML bootstrap support values (1000 replicates, normal type) above 75% are indicated at the nodes. Posterior probabilities (above 0.9) obtained from BI are indicated by bold lines at the relevant branching points. T = ex-type cultures. Scale bar = total nucleotide difference between taxa. [file 43008_2020_37_MOESM2_ESM.pptx]

## Slide 1
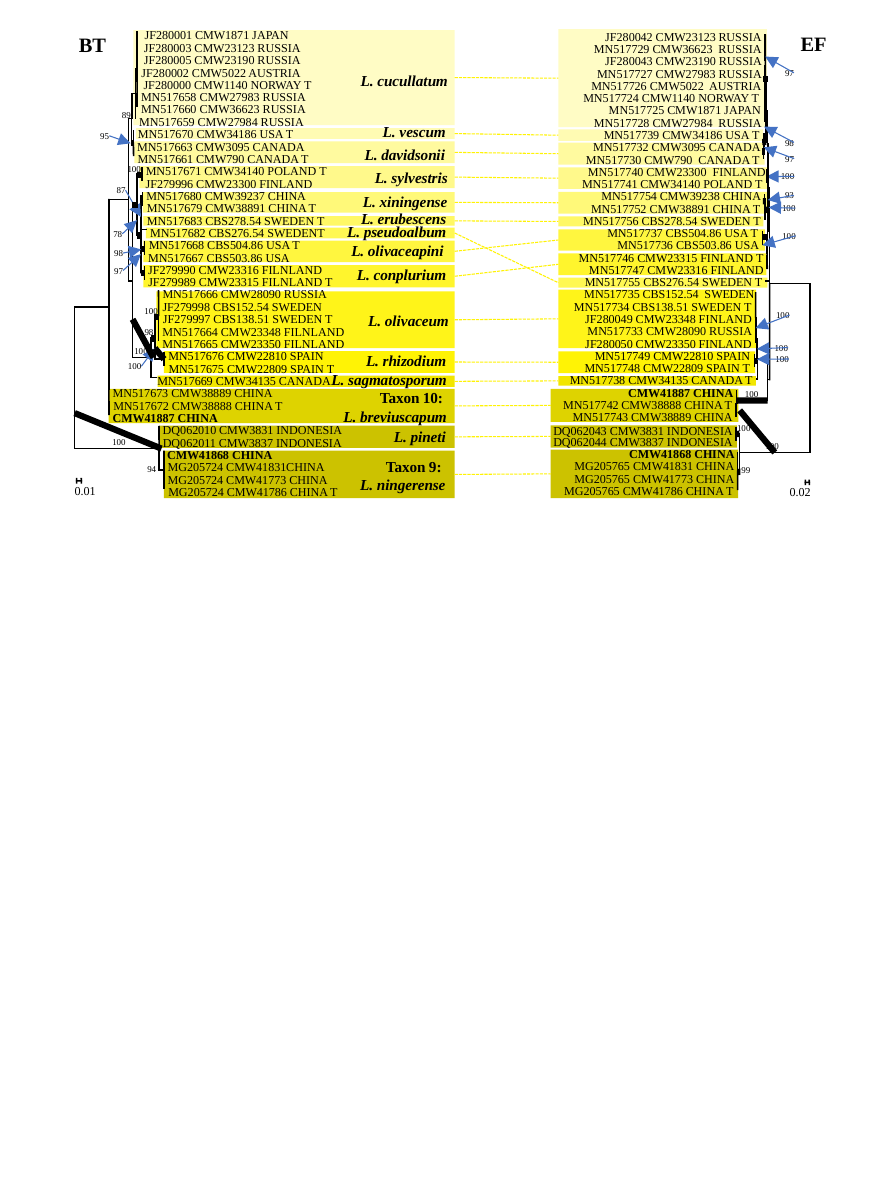

EF
BT
 JF280001 CMW1871 JAPAN
 JF280003 CMW23123 RUSSIA
 JF280005 CMW23190 RUSSIA
 JF280002 CMW5022 AUSTRIA
 JF280000 CMW1140 NORWAY T
 MN517658 CMW27983 RUSSIA
 MN517660 CMW36623 RUSSIA
89
 MN517659 CMW27984 RUSSIA
 MN517670 CMW34186 USA T
95
 MN517663 CMW3095 CANADA
 MN517661 CMW790 CANADA T
 MN517671 CMW34140 POLAND T
100
 JF279996 CMW23300 FINLAND
87
 MN517680 CMW39237 CHINA
 MN517679 CMW38891 CHINA T
 MN517683 CBS278.54 SWEDEN T
 MN517682 CBS276.54 SWEDENT
78
 MN517668 CBS504.86 USA T
98
 MN517667 CBS503.86 USA
 JF279990 CMW23316 FILNLAND
97
 JF279989 CMW23315 FILNLAND T
 MN517666 CMW28090 RUSSIA
 JF279998 CBS152.54 SWEDEN
100
 JF279997 CBS138.51 SWEDEN T
 MN517664 CMW23348 FILNLAND
98
 MN517665 CMW23350 FILNLAND
100
 MN517676 CMW22810 SPAIN
100
 MN517675 CMW22809 SPAIN T
MN517669 CMW34135 CANADA
 MN517673 CMW38889 CHINA
 MN517672 CMW38888 CHINA T
 CMW41887 CHINA
 DQ062010 CMW3831 INDONESIA
 DQ062011 CMW3837 INDONESIA
100
 CMW41868 CHINA
 MG205724 CMW41831CHINA
94
 MG205724 CMW41773 CHINA
0.01
 MG205724 CMW41786 CHINA T
JF280042 CMW23123 RUSSIA
 MN517729 CMW36623 RUSSIA
 JF280043 CMW23190 RUSSIA
 MN517727 CMW27983 RUSSIA
97
 MN517726 CMW5022 AUSTRIA
 MN517724 CMW1140 NORWAY T
 MN517725 CMW1871 JAPAN
 MN517728 CMW27984 RUSSIA
MN517739 CMW34186 USA T
98
MN517732 CMW3095 CANADA
 MN517730 CMW790 CANADA T
97
 MN517740 CMW23300 FINLAND
100
MN517741 CMW34140 POLAND T
 MN517754 CMW39238 CHINA
93
 MN517752 CMW38891 CHINA T
100
 MN517756 CBS278.54 SWEDEN T
MN517737 CBS504.86 USA T
100
MN517736 CBS503.86 USA
 MN517746 CMW23315 FINLAND T
 MN517747 CMW23316 FINLAND
MN517755 CBS276.54 SWEDEN T
 MN517735 CBS152.54 SWEDEN
 MN517734 CBS138.51 SWEDEN T
100
 JF280049 CMW23348 FINLAND
MN517733 CMW28090 RUSSIA
JF280050 CMW23350 FINLAND
100
 MN517749 CMW22810 SPAIN
100
 MN517748 CMW22809 SPAIN T
MN517738 CMW34135 CANADA T
 CMW41887 CHINA
100
MN517742 CMW38888 CHINA T
MN517743 CMW38889 CHINA
100
DQ062043 CMW3831 INDONESIA
 DQ062044 CMW3837 INDONESIA
100
 CMW41868 CHINA
 MG205765 CMW41831 CHINA
99
 MG205765 CMW41773 CHINA
 MG205765 CMW41786 CHINA T
0.02
 L. cucullatum
 L. vescum
 L. davidsonii
L. sylvestris
L. xiningense
 L. erubescens
 L. pseudoalbum
 L. olivaceapini
L. conplurium
 L. olivaceum
L. rhizodium
 L. sagmatosporum
Taxon 10:
L. breviuscapum
 L. pineti
Taxon 9:
L. ningerense
