# Supplementary material for: Ophiostomatoid fungi associated with mites phoretic on bark beetles in Qinghai, China
Source: IMA Fungus. 2020 Jul 30;11:15. doi: 10.1186/s43008-020-00037-9 (PMC7391587; doi:10.1186/s43008-020-00037-9)
Supplement: Supplementary file 3 — Additional file 3: Table S1. Ophiostomatoid fungi reported from China. [file 43008_2020_37_MOESM3_ESM.docx]

**Table S1.** Species of Ophiostomatales reported from China.

| **Fungal species** | **Location** | **Tree host** | **Beetle vector** | **Reference** |
| --- | --- | --- | --- | --- |
| *Ceratocystiopsis cf. pallidobrunnea* | Inner Mongolia | *Pinus sylvestris var. mongolica* | *Ips subelongatus* | Wang et al., 2020 |
| *Esteya vermicola* | Yunnan | *P. yunnanensis* | *Tomicus yunnanensis* | Wang et al., 2019 |
| *Graphilbum anningense* | Yunnan | *P. yunnanensis* | *T. yunnanensis*  *T. minor* | Wang et al., 2019 |
| *Gra. fragrans* | Yunnan | *P. yunnanensis*  *Tsuga dumosa*  *P. armandii* | *T. minor*  *T. yunnanensis*  *Pissodes* sp. | Paciura et al., 2010b; Zhou et al., 2013; Chang et al., 2017 |
| *Gra. kesiya* | Yunnan | *P. kesiya* | *Polygraphus szemaoensis*  *Polygraphus aterrimus*  *Polygraphus* sp. | Chang et al., 2017 |
| *Gra. puerense* | Yunnan | *P. kesiya* | *Pol. szemaoensis*  *Ips acuminatus* | Chang et al., 2017 |
| *Graphilbum cf. deltoideosporum* | Shandong | *P. massoniana* | *Monochamus alternatus/* *Bursaphelenchus xylophilus* | Wang et al., 2018 |
| *Graphilbum cf. rectangulosporium* | Zhejiang | *P. massoniana* | *M. alternatus/* *B. xylophilus* | Wang et al., 2018 |
| *Graphilbum* sp. | Shanxi | *P. tabuliformis* | *Dendroctonus valens* | Taerum et al., 2013 |
| *Grosmannia crassifolia* | Qinghai | *Picea crassifolia*  *Pi. purpurea* | *Pol. poligraphus*  *Ips shangrila* | Yin et al., 2020 |
| *G. maixiuense* | Qinghai | *Pi. crassifolia* | *Pol. poligraphus*  *I. shangrila* | Yin et al., 2020 |
| *G. piceiperda* | Jilin | *Picea* sp. | *I. typographus* | Chang et al., 2019 |
| *G. purpurea* | Qinghai | *Pi. purpurea* | *I. shangrila* | Yin et al., 2020 |
| *G. radiaticola* | Shanxi  Yunnan | *P. tabuliformis*  *Tsuga* sp. | *D. valens*  Unknown | Lu et al., 2009a; Taerum et al., 2013  Chang et al., 2017 |
| *G. xianmiense* | Qinghai | *Pi. crassifolia*  *Pi. purpurea* | *Pol. poligraphus, I. nitidus*  *I. shangrila* | Yin et al., 2020 |
| *G. yunnanensis* | Yunnan | *P. kesiya* | *Pol. Szemaoensis*  *Lasconotus* sp.  *I. acuminatus*  *Polygraphus* sp.  *Coccotrypes cyperi*  *T. brevipilosus* | Chang et al., 2017; Wang et al., 2019 |
| *G. yunnanensis* | Yunnan | *P. yunnanensis* | *T. yunnanensis* | Wang et al., 2019 |
| *Leptographium alethinum* | Shanxi | *P. tabuliformis* | *D. valens* | Lu et al., 2009a; Taerum et al., 2013 |
| *L. altius* | Jilin | *L. olgensis*  *P. koraiensis* | *I. cembrae* | Paciura et al., 2010a |
| *L. bachii* | Heilongjiang | *P. koraiensis* | *I. typographus* | Chang et al., 2019 |
| *L. breviuscapum* | Qinghai | *Pi. crassifolia* | *Pol. poligraphus* | Yin et al., 2019 |
| *L. celere* | Yunnan  Jilin | *P. kesiya*  *Pinus* sp. | – | Paciura et al., 2010a |
| *L. conjunctum* | Yunnan | *P. yunnanensis*  *P. kesiya* | *Hylurgops major* | Paciura et al., 2010a; Zhou et al., 2013; Chang et al., 2017 |
| *L. cucullatum* | Heilongjiang | *P. koraiensis* | *I. typographus* | Chang et al., 2019 |
| *L. curviconidium* | Jilin | *P. koraiensis* | *I. typographus* | Paciura et al., 2010a |
| *L. duchongii* | Heilongjiang | *P. koraiensis* | *I. typographus* | Chang et al., 2019 |
| *L. fenglinhensis* | Heilongjiang | *P. koraiensis* | *I. typographus* | Chang et al., 2019 |
| *L. gracile* | Yunnan | *P. armandii* | *Pissodes* sp. | Paciura et al., 2010a; Zhou et al., 2013; Chang et al., 2017 |
| *L. heilongjiangii* | Heilongjiang | *P. koraiensis* | *I. typographus* | Chang et al., 2019 |
| *L. innermongolicum* | Inner Mongolia | *L. gmelinii* | *I. subelongatus* | Liu et al., 2017 |
| *L. koreanum* | Shanxi  Shannxi | *P. tabuliformis* | *D. valens* | Lu et al., 2009a; Taerum et al., 2013 |
| *L. manifestum* | Jilin  Yunnan | *L. olgensis*  *P. yunnanensis* | *I. subelongatus*  *P. verrucifrons* | Paciura et al., 2010a |
| *L. ningerense* | Yunnan | *P. kesiya* | *Co. cyperi*  *Orthotomicus angulatus* | Chang et al., 2017 |
| *L. olivaceum* | Heilongjiang | *P. koraiensis* | *I. typographus* | Chang et al., 2019 |
| *L. penicillata* | Heilongjiang | *P. koraiensis* | *I. typographus* | Chang et al., 2019 |
| *L. pineti* | Yunnan | *P. kesiya* | – | Paciura et al., 2010a |
| *L. pinicola* | Shanxi | *P. tabuliformis* | *D. valens* | Lu et al., 2009a; Taerum et al., 2013 |
| *L. pini-densiflorae* | Shanxi  Shannxi | *P. tabuliformis* | *D. valens* | Lu et al., 2009a; Taerum et al., 2013 |
| *L. pistaciae* | Yunnan | *Pistacia chinensis* | – | Paciura et al., 2010a |
| *L. procerum* | Shanxi  Shannxi  Henan | *P. tabuliformis*  *P. bungeana* | *D. valens* | Lu et al., 2009a; Lu et al., 2009b; Taerum et al., 2013 |
| *L. shanshensis* | Heilongjiang | *P. koraiensis* | *I. typographus* | Chang et al., 2019 |
| *L. sinense* | Jiangxi | *P. elliottii* | *Hylobitelus xiaoi* | Yin et al., 2015 |
| *L. sinoprocerum* | Shanxi  Hebei | *P. tabuliformis*  *P. bungeana* | *D. valens* | Lu et al., 2009a; Lu et al., 2009b; Taerum et al., 2013 |
| *L. taigense* | Inner Mongolia | *L. gmelinii* | *I. subelongatus* | Liu et al., 2017 |
| *L. truncatum* | Shanxi | *P. tabuliformis* | *D. valens* | Lu et al., 2009a; Lu et al., 2009b; Taerum et al., 2013 |
| *L. xiningense* | Qinghai | *Pi. crassifolia* | *Pol. poligraphus* | Yin et al., 2019 |
| *L. yichunensis* | Heilongjiang | *P. koraiensis* | *I. typographus* | Chang et al., 2019 |
| *L. zhangii* | Heilongjiang | *L. gmelinii* | *I. subelongatus* | Liu et al., 2017; Wang et al., 2020 |
| *O. acarorum* | Yunnan | *P. kesiya* | *I. acuminatus*  *Pol. szemaoensis*  *Cy. luteus*  *O. angulatus*  *Co. cyperi* | Chang et al., 2017 |
| *O. aggregatum* | Yunnan | *P. yunnanensis*  *P. kesiya* | *T. minor*  *T. yunnanensis* | Wang et al., 2019 |
| *O. ainoae* | Jilin  Heilongiang | *Picea* sp.  *Pi. koraiensis* | *I. typographus* | Chang et al., 2019 |
| *O. album* | Zhejiang | *P. massoniana* | *M. alternatus/* *B. xylophilus* | Wang et al., 2018 |
| *O. bicolor* | Heilongjiang | *Pi. koraiensis* | *I. typographus* | Chang et al., 2019 |
| *O. brevipilosi* | Yunnan | *P. kesiya* | *T. brevipilosus* | Chang et al., 2017; Wang et al., 2019 |
| *O. brunneolum* | Heilongjiang | *Pi. koraiensis* | *I. typographus* | Chang et al., 2019 |
| *O. canum* | Yunnan | *P. yunnanensis* | *T. minor* | Wang et al., 2019 |
| *O. floccosum* | Shanxi | *P. tabuliformis* | *D. valens* | Lu et al., 2009a; Lu et al., 2009b; Taerum et al., 2013 |
| *O. genhense* | Inner Mongolia | *Larix gmelinii* | *I. subelongatus* | Wang et al., 2020 |
| *O. hongxingense* | Helongjiang | *L. gmelinii* | *I. subelongatus* | Wang et al., 2020 |
| *O. ips* | Shanxi  Yunnan | *P. kesiya*  *P. tabuliformis* | *I. acuminatus*  *Pol. szemaoensis*  *Polygraphus* sp.  *D. valens* | Lu et al., 2009a; Taerum et al., 2013; Zhou et al., 2013; Chang et al., 2017 |
| *O. ips* | Zhejiang  Shandong | *P. massoniana*  *P. thunbergii* | *M. alternatus/* *B. xylophilus* | Wang et al., 2018 |
| *O. japonicum* | Heilongjiang | *Pi. koraiensis* | *I. typographus* | Chang et al., 2019 |
| *O. jiamusi* | Jilin | *Picea* sp. | *I. typographus* | Chang et al., 2019 |
| *O. jilinensis* | Jilin | *Picea* sp. | *I. typographus* | Chang et al., 2019 |
| *O. lotiforme* | Inner Mongolia | *P. sylvestris var. mongolica* | *I. subelongatus* | Wang et al., 2020 |
| *O. massoniana* | Zhejiang | *P. massoniana* | *M. alternatus/* *B. xylophilus* | Wang et al., 2018 |
| *O. micans* | Qinghai | *Pi. crassifolia* | *D. micans* | Yin et al., 2016 |
| *O. minus* Europe | Inner Mongolia | *L. gmelinii*  *P. sylvestris var. mongolica* | *I. subelongatus* | Wang et al., 2020 |
| *O. minus* Europe | Shanxi | *P. tabuliformis* | *D. valens* | Lu et al., 2009a; Taerum et al., 2013 |
| *O. minus* Europe | Yunnan | *P. yunnanensis* | *T. yunnanensis* | Wang et al., 2019 |
| *O. multisynnematum* | Inner Mongolia | *L. gmelinii* | *I. subelongatus* | Wang et al., 2020 |
| *O. nitidum* | Qinghai | *Pi. crassifolia* | *I. nitidus* | Yin et al., 2016 |
| *O. olgensis* | Heilongjiang  Inner Mongolia | *L. gmelini*  *L. olgensis* | *I. subelongatus* | Wang et al., 2016 |
| *O. peniculi* | Inner Mongolia | *L. gmelinii* | *I. subelongatus* | Wang et al., 2020 |
| *O. piceae* | Jilin  Shanxi | *P. tabuliformis*  *L. olgensis* | *D. valens*  *I. subelongatus* | Lu et al., 2009a; Paciura et al., 2010b; Taerum et al., 2013 |
| *O. poligraphi* | Qinghai | *Pi. crassifolia* | *P. poligraphus* | Yin et al., 2016 |
| *O. pseudobicolor* | Inner Mongolia  Heilongjiang | *L. gmelinii*  *L. principis-rupprechtii* | *I. subelongatus* | Wang et al., 2020 |
| *O. qinghaiense* | Qinghai | *Pi. crassifolia* | *P. poligraphus*  *D. micans* | Yin et al., 2016 |
| *O. quercus* | Yunnan | *P. yunnanensis*  *Abies* sp.  *P. semaonensis*  *Tsuga* sp.  *P. kesiya*  *T. dumosa*  *S. babylonica* | *I. acuminatus*  *Pol. szemaoensis*  *Pol. verrucifrons*  *T. piniperda*  Unknown spp.  *Pissodes* sp. | Paciura et al., 2010b; Zhou et al., 2013; Chang et al., 2017 |
| *O. rufum* | Inner Mongolia | *L. gmelinii* | *I. subelongatus* | Wang et al., 2020 |
| *O. setosum* | Yunnan | *Tsuga* sp.  *Abies* sp.  *T. dumosa* | Unknown  *Pissodes* sp. | Paciura et al., 2010b; Zhou et al., 2013; Chang et al., 2017 |
| *O. shangrilae* | Qinghai | *Pi. purpurea* | *I. shangrila* | Yin et al., 2016 |
| *O. subelongati* | Heilongjiang | *L. gmelinii* | *I. subelongatus* | Wang et al., 2020 |
| *O. tingens* | Yunnan | *P. yunnanensis* | *T. yunnanensis*  *T. minor* | Wang et al., 2019 |
| *O. tsotsi* | Yunnan | *P. kesiya* | *Co. cyperi*  *Cyrtogenius Luteus* | Chang et al., 2017 |
| *O. typographus* | Heilongjiang | *P. koraiensis* | *I. typographus* | Chang et al., 2019 |
| *O. wuyingii* | Heilongjiang | *P. koraiensis* | *I. typographus* | Chang et al., 2019 |
| *O. xinganense* | Inner Mongolia | *L. gmelinii* | *I. subelongatus* | Wang et al., 2020 |
| *O. yunshanensis* | Heilongjiang | *P. koraiensis* | *I. typographus* | Chang et al., 2019 |
| *Ophiostoma* sp. 1 | Jilin  Yunnan | *L. olgensis*  *T. dumosa* | *I. subelongatus*  *Pissodes* sp. | Zhou et al., 2013 |
| *Ophiostoma* sp. 2 | Jilin | *L. olgensis* | *I. subelongatus* | Zhou et al., 2013 |
| *Ophiostoma* sp. 3 | Shanxi | *P. tabuliformis* | *D. valens* | Lu et al., 2009a; Taerum et al., 2013 |
| *Ophiostoma* sp. 4 | Jilin | *P. koraiensis* | – | Zhou et al., 2013 |
| *Ophiostoma* sp. 5 | Yunnan | *P. yunnanensis*  *Abies* sp.  *P. kesiya* | *H. major* | Zhou et al., 2013 |
| *Ophiostoma* sp. 6 | Yunnan | *P. semaonensis* | Unknown | Chang et al., 2017 |
| *Ophiostoma* sp. 7 | Yunnan | *Tsuga* sp. | Unknown | Chang et al., 2017 |
| *Sporothrix abietina* | Shanxi | *P. tabuliformis* | *D. valens* | Lu et al., 2009a; Taerum et al., 2013 |
| *S. macroconidia* | Yunnan | *P. yunnanensis*  *P. kesiya* | *T. yunnanensis*  *T. brevipilosus* | Wang et al., 2019 |
| *S. nebularis* | Yunnan | *P. yunnanensis*  *P. kesiya* | *T. piniperd*  *Co. cyperi*  *Cy. luteus*  *Or. angulatus*  *Polygraphus* sp. | Chang et al., 2017 |
| *S. pseudoabietina* | Yunnan | *P. yunnanensis* | *T. minor* | Wang et al., 2019 |
| *S. zhejiangensis* | Zhejiang | *P. massoniana* | *M. alternatus/* *B. xylophilus* | Wang et al., 2018 |
| *Sporothrix* sp. A | Yunnan | *P. kesiya* | *Co. cyperi* | Chang et al., 2017 |

**References**

Chang R, Duong TA, Taerum SJ, Wingfield MJ, Zhou XD, De Beer ZW (2017) Ophiostomatoid fungi associated with conifer-infesting beetles and their phoretic mites in Yunnan, China. MycoKeys 28: 19-64.

Chang R, Duong TA, Taerum SJ, Wingfield MJ, Zhou XD, De Beer ZW (2019) Ophiostomatoid fungi associated with the spruce bark beetle *Ips typographus*, including 11 new species from China. Persoonia 42: 50-74.

Liu X-W, Wang H-M, Lu Q, Decock C, Li Y-X, Zhang X-Y (2017) Taxonomy and pathogenicity of *Leptographium* species associated with *Ips subelongatus* infestations of *Larix* spp. in northern China, including two new species. Mycological Progress 16: 1-13.

Lu M, Zhou XD, De Beer ZW, Wingfield MJ, Sun J-H (2009a) Ophiostomatoid fungi associated with the invasive pine-infesting bark beetle, *Dendroctonus valens,* in China. Fungal Diversity 38: 133-145.

Lu Q, Decock C, Zhang X, Maraite H (2009b) Ophiostomatoid fungi (Ascomycota) associated with *Pinus tabuliformis* infested by *Dendroctonus valens* (Coleoptera) in northern China and an assessment of their pathogenicity on mature trees. Antonie van Leeuwenhoek 96: 275-293.

Paciura D, De Beer ZW, Jacobs K, Zhou XD, Ye H, Wingfield MJ (2010a) Eight new *Leptographium* species associated with tree-infesting bark beetles in China. Persoonia 25: 94-108.

Paciura D, Zhou XD, De Beer ZW, Jacobs K, Ye H, Wingfield M (2010b) Characterisation of synnematous bark beetle-associated fungi from China, including *Graphium carbonarium* sp. nov. Fungal Diversity 40: 75-88.

Taerum SJ, Duong TA, De Beer ZW, Gillette N, Sun J-H, Owen DR, Wingfield MJ (2013) Large shift in symbiont assemblage in the invasive red turpentine beetle. PLoS ONE 8: e78126.

Wang H-M, Lu Q, Meng X-J, Liu X-W, Decock C, Zhang X-Y (2016) *Ophiostoma olgensis*, a new species associated with *Larix* spp. and *Ips subelongatus* in northern China. Phytotaxa 282: 282-290.

Wang H, Lun Y, Lu Q, Liu H, Decock C, Zhang X (2018) Ophiostomatoid fungi associated with pines infected by *Bursaphelenchus xylophilus* and *Monochamus alternatus* in China, including three new species. MycoKeys 39: 1-27.

Wang H, Wang Z, Liu F, Wu CX, Zhang SF, Kong Xiang B, Decock C, Lu Q, Zhang Z (2019) Differential patterns of ophiostomatoid fungal communities associated with three sympatric *Tomicus* species infesting pines in south-western China, with a description of four new species. MycoKeys 50: 93-133.

Wang Z, Liu Y, Wang H, Meng X, Liu X, Decock C, Zhang X, Lu Q (2020) Ophiostomatoid fungi associated with *Ips subelongatus*, including eight new species from northeastern China. IMA Fungus 11: 3.

Yin M, Duong T, Wingfield M, Zhou X, De Beer ZW (2015) Taxonomy and phylogeny of the *Leptographium procerum* complex, including *Leptographium sinense* sp. nov. and *Leptographium longiconidiophorum* sp. nov. Antonie van Leeuwenhoek 107: 547-563.

Yin M, Wingfield MJ, Zhou X, De Beer ZW (2016) Multigene phylogenies and morphological characterization of five new *Ophiostoma* spp. associated with spruce-infesting bark beetles in China. Fungal Biology 120: 454–470.

Yin M, Wingfield MJ, Zhou X, De Beer ZW (2020) Phylogenetic re-evaluation of the *Grosmannia penicillata* complex (Ascomycota, Ophiostomatales), with the description of five new species from China and USA. *Fungal Biology*. **124:** 110-124.

Yin M, Wingfield MJ, Zhou X, Linnakoski R, De Beer ZW (2019) Taxonomy and phylogeny of the *Leptographium olivaceum* complex (Ophiostomatales, Ascomycota), including descriptions of six new species from China and Europe. *MycoKeys*. **60:** 93-123.

Zhou X, Song RQ, Zhou XD, Cui L, Cao C (2011) Fungal population in the inside and outside of *Ips subelongatus* body and the gallary of insect-bored larch logs. Mycosystema 30: 400-407.

Zhou XD, De Beer ZW, Wingfield MJ (2013) Ophiostomatoid fungi associated with conifer-infesting bark beetles in China. In: The Ophiostomatoid Fungi: Expanding Frontiers (KA Seifert, ZW De Beer & MJ Wingfield, eds): 3-100. CBS-KNAW Biodiversity Centre, Utrecht, The Netherlands.
