# Supplementary material for: Ophiostomatoid fungi associated with mites phoretic on bark beetles in Qinghai, China
Source: IMA Fungus. 2020 Jul 30;11:15. doi: 10.1186/s43008-020-00037-9 (PMC7391587; doi:10.1186/s43008-020-00037-9)
Supplement: Supplementary file 4 — Additional file 4: Table S2. Numbers of mite individuals collected and numbers of mite individuals carring fungi in this study. [file 43008_2020_37_MOESM4_ESM.docx]

**Table S2.** Numbers of mite individuals were collected and numbers of mite individuals carrying fungi in this study.

| **Beetle**  **species** | **Mite species^1^**^, 2^ | | | | | | | | | | | | | | | | | | | | | | | | | | | | | | | | | | | | | | | | | | | | | | | |
| --- | --- | --- | --- | --- | --- | --- | --- | --- | --- | --- | --- | --- | --- | --- | --- | --- | --- | --- | --- | --- | --- | --- | --- | --- | --- | --- | --- | --- | --- | --- | --- | --- | --- | --- | --- | --- | --- | --- | --- | --- | --- | --- | --- | --- | --- | --- | --- | --- |
|  | **M1** | | **M2** | | **M3** | | **M4** | | **M5** | | **M6** | | **M7** | | **M8** | | **M9** | | **M10** | | **M11** | | **M12** | | **M13** | | **M14** | | **M15** | | **M16** | | **M17** | | **M18** | | **M19** | | **M20** | | **M21** | | **M22** | | **M23** | | **M24** | |
|  | **A** | **B** | **A** | **B** | **A** | **B** | **A** | **B** | **A** | **B** | **A** | **B** | **A** | **B** | **A** | **B** | **A** | **B** | **A** | **B** | **A** | **B** | **A** | **B** | **A** | **B** | **A** | **B** | **A** | **B** | **A** | **B** | **A** | **B** | **A** | **B** | **A** | **B** | **A** | **B** | **A** | **B** | **A** | **B** | **A** | **B** | **A** | **B** |
| *D. micans* | 0 | 0 | 0 | 0 | 3 | 1 | 0 | 0 | 0 | 0 | 0 | 0 | 0 | 0 | 0 | 0 | 0 | 0 | 0 | 0 | 1 | 1 | 0 | 0 | 0 | 0 | 0 | 0 | 0 | 0 | 0 | 0 | 0 | 0 | 0 | 0 | 0 | 0 | 0 | 0 | 0 | 0 | 0 | 0 | 9 | 9 | 0 | 0 |
| *I. nitidus* | 0 | 0 | 2 | 2 | 0 | 0 | 1 | 0 | 23 | 7 | 11 | 3 | 3 | 2 | 0 | 0 | 6 | 1 | 10 | 5 | 0 | 0 | 0 | 0 | 1 | 1 | 0 | 0 | 0 | 0 | 5 | 1 | 0 | 0 | 29 | 8 | 0 | 0 | 21 | 6 | 0 | 0 | 0 | 0 | 2 | 1 | 1 | 1 |
| *I. shangrila* | 1 | 0 | 0 | 0 | 0 | 0 | 0 | 0 | 0 | 0 | 2 | 2 | 0 | 0 | 1 | 0 | 0 | 0 | 0 | 0 | 0 | 0 | 0 | 0 | 0 | 0 | 0 | 0 | 6 | 1 | 0 | 0 | 7 | 1 | 6 | 5 | 0 | 0 | 11 | 1 | 1 | 1 | 1 | 0 | 0 | 0 | 0 | 0 |
| *P. polygraphus* | 0 | 0 | 0 | 0 | 0 | 0 | 0 | 0 | 0 | 0 | 2 | 2 | 0 | 0 | 0 | 0 | 0 | 0 | 0 | 0 | 0 | 0 | 1 | 0 | 0 | 0 | 2 | 1 | 0 | 0 | 0 | 0 | 0 | 0 | 0 | 0 | 1 | 0 | 3 | 2 | 0 | 0 | 0 | 0 | 0 | 0 | 0 | 0 |

^1^ Mite species see Table 1

^2^ A = Number of mite individuals in beetle galleries; B = Number of mite individuals carrying fungi
